# Glycine Composition and Ion Valency Tune Phase Behavior and Drug Encapsulation in Designer Peptide Condensates

**DOI:** 10.1021/acsami.5c19632

**Published:** 2026-02-12

**Authors:** Shirel Veretnik, Rif Harris, Ayala Lampel

**Affiliations:** † Shmunis School of Biomedicine and Cancer Research, George S. Wise Faculty of Life Sciences, 26745 Tel Aviv University, Tel Aviv 6997801, Israel; ‡ Center for Nanoscience and Nanotechnology Tel Aviv University, Tel Aviv 6997801, Israel; § Center for Physics and Chemistry of Living Systems Tel Aviv University, Tel Aviv 6997801, Israel; ∥ Leibniz Institute of Polymer Research Dresden Max Bergmann Center of Biomaterials Dresden, 01069 Dresden, Germany

**Keywords:** biomolecular condensates, liquid−liquid phase separation, peptides, coacervates, drug encapsulation

## Abstract

Nano- and microencapsulation that combines high loading capacity with stimulus-responsive release remains a challenge for therapeutic delivery. Designed peptide condensates formed by liquid–liquid phase separation offer a versatile and biocompatible platform to address this need. Here, we systematically link backbone flexibility and ion identity to phase behavior, material properties, payload encapsulation, and protease-triggered condensate disassembly using minimalistic cationic–aromatic peptides that differ in their glycine content. Moreover, we systematically studied how monovalent vs divalent anions affect phase behavior and payload encapsulation. Our results show that glycine-poor sequence forms the most highly packed condensates and that the kosmotrope divalent sulfate ions markedly increase dense-phase peptide concentration and droplet size. Glycine content, which regulates charge density and backbone flexibility, directly affects condensate dynamics, showing faster diffusion with an increasing number of glycine residues. High-performance liquid chromatography partitioning analysis of five FDA-approved small molecules demonstrates compound-specific and salt-mediated recruitment, where both the hydrophobicity/polarity and the charge state of the compounds affect their encapsulation in the dense phase. Moreover, using trypsin as a proteolytic trigger, we show how the glycine content affects condensate disassembly. Overall, these results facilitate practical design rules that show how to tune charge and aromatic density, backbone flexibility, and ion valency to regulate dense-phase packing, payload encapsulation, and release. These insights advance the rational engineering of peptide condensates for targeted sequestration and controlled therapeutic release.

## Graphical abstract

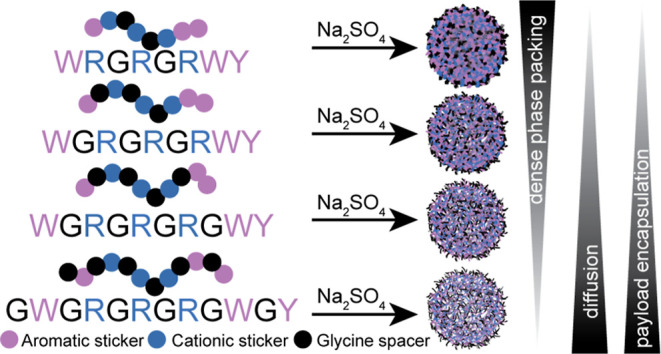

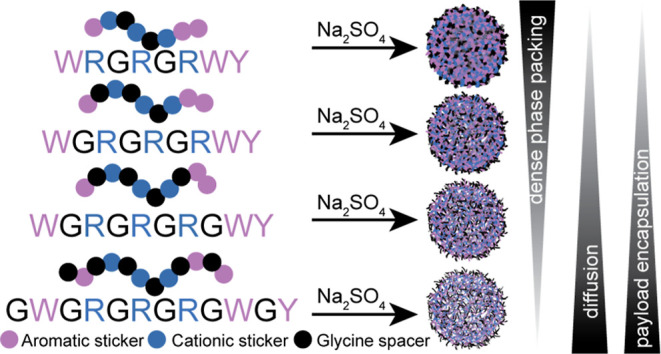

## Introduction

Nano- and microencapsulation of therapeutic (bio)­molecules remains a central challenge at the interface of materials science and medicine. Over decades, a wide variety of carriers, including lipid nanoparticles, polymersomes, inorganic nanoparticles, and protein/DNA cages, have been developed to protect cargos, improve pharmacokinetics, and enable controlled delivery to target tissues. These architectures have led to major translational successes,^1–5^ yet they also hold major limitations as many solid-state carriers require energy-intensive fabrication, display limited capacity for large or highly charged payloads, and can impose substantial diffusion barriers that restrict triggered release at the target site. As a result, the search for alternative, biocompatible compartments that combine a high loading capacity with tunable responsiveness and facile synthesis remains urgent.

The discovery and mechanistic characterization of liquid–liquid phase separation (LLPS) in biology, which underlies the formation of membraneless organelles, also known as biomolecular condensates, have inspired a new materials paradigm for the design of microcompartments. Unlike rigid nanoparticles, condensates are dynamic, liquid-like phases that concentrate biomolecules locally while permitting rapid exchange with the surrounding medium. These properties are advantageous for applications that require both sequestration and on-demand release, and they have already been exploited to create enzyme-rich microreactors,^6–9^ sensing platforms,^10,11^ and delivery systems.^12–15^ Synthetic condensates can be designed and constructed from the bottom up by a wide range of building blocks,^16^ including engineered polypeptides,^17^ minimalistic peptides,^15,18–23^ and DNA^24^ or RNA polymers.^25–27^ The formation of both biological and synthetic condensates is governed by multiple multivalent intra- and intermolecular attractive forces^28^ including H-bonding, electrostatic, π–π, and cation–π interactions that collectively induce the formation of two immiscible phases, polymer-rich dense phase and polymer-poor dilute phase.^29^ These multivalent interactions together determine the formation, composition, and material properties of the dense phase. This tunability opens opportunities to engineer condensates with application-specific capacities that complement, or in some cases outperform, traditional carriers and compartments.

Among the various building blocks, peptides represent an especially attractive scaffold for designed condensates. Their sequence programmability,^30,31^ ease of synthesis and purification, and biocompatible chemistry allow precise control over multivalency and specific side-chain interactions.^18,19,23^ Recent studies have shown that minimal peptide motifs can drive robust LLPS and be harnessed as enzymatic microreactors^7,8,32^ and simple delivery vehicles.^13^ Compared with intrinsically disordered proteins, short peptides reduce sequence and structural complexity and enable straightforward structure–function mapping at the sequence level,^30^ making them excellent model systems for deriving practical design rules for condensate-based materials.

Despite these advantages, fundamental gaps remain in understanding how simple sequence modifications translate into the macroscopic properties that are required for the encapsulation of therapeutic payloads. Specifically, it is still unknown which sequence features control the dense-phase packing, internal fluidity, payload partitioning, and condensate disassembly. Prior work has identified general roles for charge patterning,^33^ aromatic content,^34^ and hydrophobicity,^35,36^ and a growing literature reports the importance of ionic composition, both in terms of ion concentration and ion identity, in modulating phase diagrams and condensate material properties.^36–40^ Although a large number of studies describe salt-dependent effects, there is a need for systematic studies that relate ion-mediated dense-phase enrichment to practical outcomes for drug partitioning.

In this work, we sought to establish connections that link charge and aromatic density and backbone flexibility, provided by the number of glycine residues within minimalistic peptides, to LLPS propensity, dense-phase peptide concentration, diffusivity, payload encapsulation, and protease-induced disassembly. The small side chain of glycine influences the backbone conformational freedom and local ordering. Varying glycine content in short cationic–aromatic peptides therefore provides a means to tune backbone flexibility and, in turn, the balance between chain mobility and intermolecular packing. Moreover, we aimed to elucidate how ion composition, and specifically how monovalent vs divalent anions, affects phase separation properties and small-molecule encapsulation. To address these questions systematically, we designed four minimalistic LLPS-promoting peptides that have the same aromatic and cationic amino acid compositions but differ in the number of glycine residues. Our findings show that modest changes in glycine content result in significant and systematic effects on phase behavior. A glycine-poor sequence forms denser and more slowly diffusing condensates compared to a glycine-rich peptide, which forms fluid phases that favor higher encapsulation of composition-dependent hydrophobic drugs. We also found that the strong kosmotrope divalent sulfate ions significantly promote dense-phase packing and enhance the encapsulation of hydrophobic small-molecule therapeutics by dehydration and increasing local peptide concentration. Intermediate glycine composition provides an optimal balance between efficient payload encapsulation and protease-triggered release, thus offering practical design rules for tuning peptide condensates for delivery applications.

## Results and Discussion

### Glycine Content and Salt Composition Affect LLPS Properties

To study how the glycine content modulates phase behavior in minimal LLPS-promoting peptides, we designed four peptides that maintain the same number of aromatic and cationic residues but differ in their glycine content (Figure 1). Each sequence contains three arginines and three aromatics, two tryptophans, and one tyrosine, while the number of glycines is systematically varied. This minimalistic LLPS motif (three arginines and three aromatics) is based on previously validated sequence frameworks shown to reliably undergo simple coacervation.^20^ Moreover, inserting glycine residues between charged and aromatic stickers (rather than in a continuous block) maintains the sticker–spacer architecture^41^ characteristic of the known LLPS-forming peptides and allows us to systematically tune backbone flexibility without altering sticker patterning. G2 (WRGRGRWY) contains only two glycine residues, G3 (WGRGRGRWY) contains three glycine residues, G4 (WGRGRGRGWY) contains four glycine residues, and G6 (GWGRGRGRGWGY) contains six glycine residues. Since ionic strength and ion valency strongly influence coacervation, especially in the current minimalistic system which relies on simple coacervation of cationic peptides with a net charge of (+3), we compared phase behavior in the presence of two salts, the first containing a monovalent anion (NaCl) and the second salt containing a divalent strong kosmotrope anion (Na_2_SO_4_).

**Figure 1:**
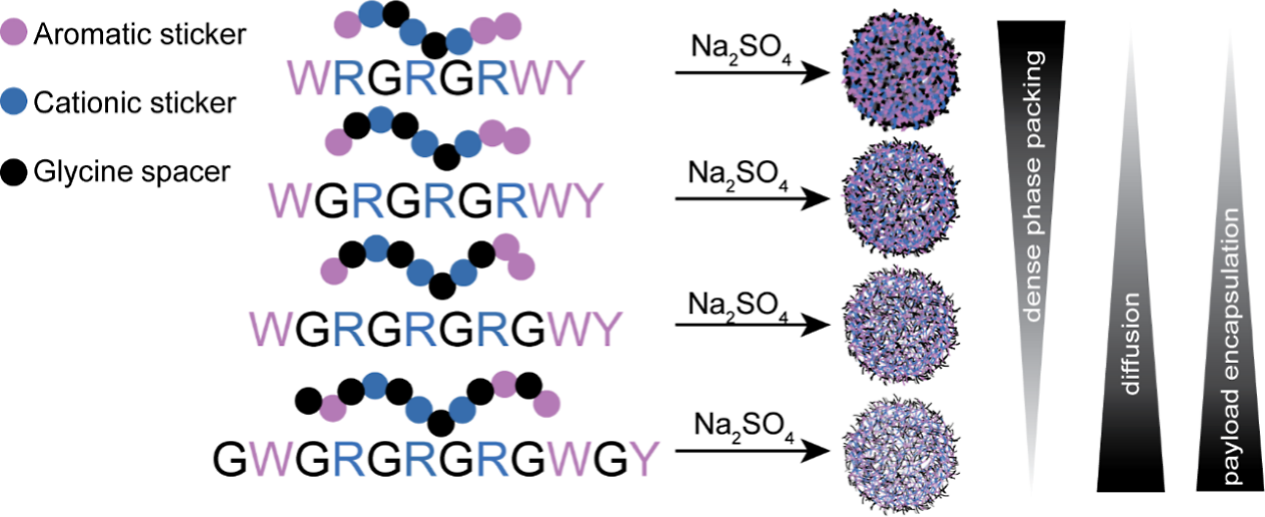
Glycine content and divalent anions regulate dense-phase packing, diffusivity, and payload encapsulation. Schematic illustration of the designed minimalistic peptide sequences (represented using a single-letter code, where each amino acid is represented as a sphere) containing the aromatic residues tryptophan and tyrosine (purple), three arginine residues (blue), and varying number of glycine residues (black). Increasing the number of glycine residues within the peptide sequence and thus decreasing the aromatic and charge density and increasing backbone disorder decreases the dense-phase packing and increases the dense-phase diffusivity and the encapsulation of small-molecule therapeutic payloads.

We first conducted microscopy analysis to characterize the LLPS propensity of the peptides. For this, each peptide was dissolved in phosphate buffer, and the pH was adjusted to 7.5. NaCl or Na_2_SO_4_ was added to each sample at varying concentrations to study the effect of mono- vs divalent anions on phase behavior. We then compared the abundance of condensate formation of the four peptides as a factor of peptide and salt concentrations as well as salt composition (NaCl vs Na_2_SO_4_). This analysis reveals two clear effects: (i) the glycine content shifts the peptide saturation concentration (*C*_sat_), and (ii) the preferred salt species modulates which sequence is most LLPS-prone (Figure 2). In NaCl, the glycine-poor peptides G2 and G3 show the lowest *C*_sat_ (10 mM), indicating the highest LLPS propensity (Figures 2a and S1). Yet, in Na_2_SO_4_, the intermediate glycine peptides G3 and G4 display the lowest *C*_sat_ (5 mM) (Figure 2). Notably, at 20 mM in salt-free conditions, LLPS was observed for G2, G3, and G4, while G6 undergoes aggregation (Figure S2). We hypothesize that this aggregation arises not from the increased glycine content per se, as increasing backbone flexibility is not expected to result in aggregation, but from the specific sequence arrangement: the glycine residue at the first position and the tryptophan residue at the second position. To test this, we examined three additional peptides, previously reported by us,^8,20^ which contain six glycine residues and place tryptophan at the first position (WGRGRGRGWPGVGY, termed WGR-1, WGRGRGRGWQGVGY, termed WGR-Q, and WGRGRGRGWPGSGY, termed WGR-S). As expected, all three peptides formed typical spherical droplets in both the absence and presence of 2.5 mM and 5 mM Na_2_SO_4_, and no aggregates were observed (Figure S3). These results suggest that the aggregation observed for G6 in the absence of Na_2_SO_4_ can be attributed to the position of the G residue rather than to the glycine content alone.

**Figure 2:**
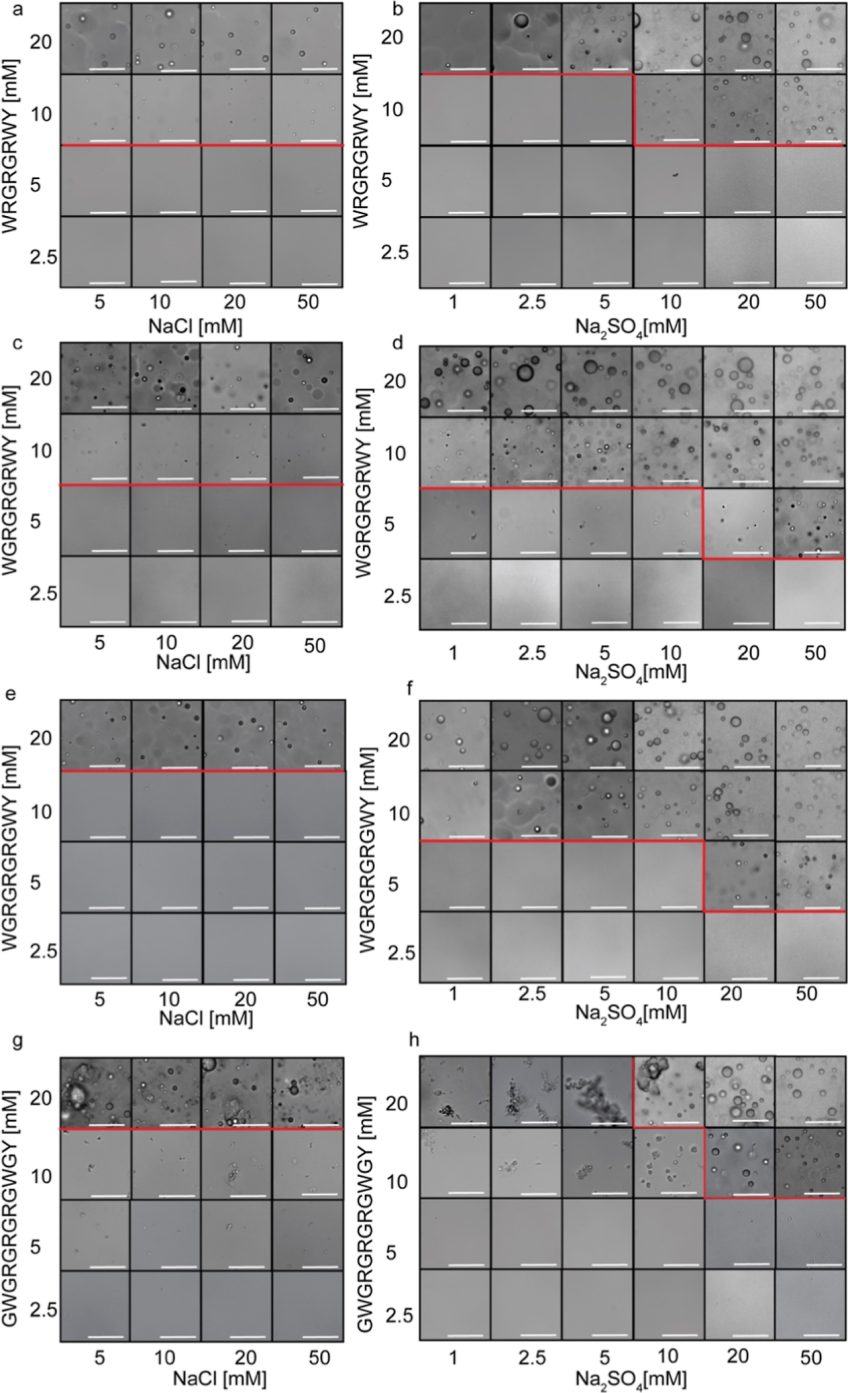
Glycine content and salt composition affect LLPS propensity. Phase diagrams obtained by bright-field microscopy analysis of the peptides G2 (a,b), G3 (c,d), G4 (e,f), and G6 (g,h) as a function of peptide, NaCl, and Na_2_SO_4_ concentrations. Peptides were dissolved in 20 mM phosphate buffer pH 7.5, and salt or double-distilled water (ddw) was added subsequently. Scale bar = 25 μm.

We also tested how substituting Y with phenylalanine (F) affects LLPS propensity. By focusing on G4 as a model, we designed the peptide G4F that has a Y/F substitution. In NaCl, the peptide G4F showed higher LLPS propensity, with lower *C*_sat_ (10 mM) compared to G4 (20 mM). In Na_2_SO_4_, G4 underwent LLPS at a peptide concentration of 5 mM with 20 mM Na_2_SO_4_, whereas G4F phase separated at 5 mM at a higher ionic strength of 50 mM NaSO_4_ (Figures S4 and 2e). This result also correlates with our prior study^20^ showing that both Y and F side chains strongly contribute to intermolecular interactions with the side chain of R. Notably, we also observed that phosphate buffer itself contributes to the homotypic LLPS of the cationic peptide owing to its charge state at pH 7.5 ^20^ compared to Tris buffer at pH 7.5 (Figure S5).

### Effect of Ion Composition on the Condensate Size and Dense-Phase Peptide Concentration

To characterize how glycine content and salt composition affect the condensate diameter, we performed dynamic light scattering (DLS) analysis of each peptide at 20 mM with and without NaCl and Na_2_SO_4_ (20 mM). The results of the DLS analysis correlate with the microscopy observations (Figure 3a–d). In salt-free buffer, G4 and G6 produce assemblies (∼0.7 and 2 μm) larger than those of G2 and G3. Addition of NaCl and even more so, of Na_2_SO_4_, shifts the population toward larger, micron-scale condensates (∼1.5–6 μm) for G2, G3, and G4. G6 shows high polydispersity, consistent with the presence of aggregates and mixed populations. The DLS results correlate with the measured diameter based on the bright-field microscopy analysis (Figure S6). Taken together, these results indicate that the kosmotrope divalent anions promote the formation of larger dense phases. Similar observations were previously reported with nitrates, which were found to promote phase separation compared to chlorides.^38^

**Figure 3:**
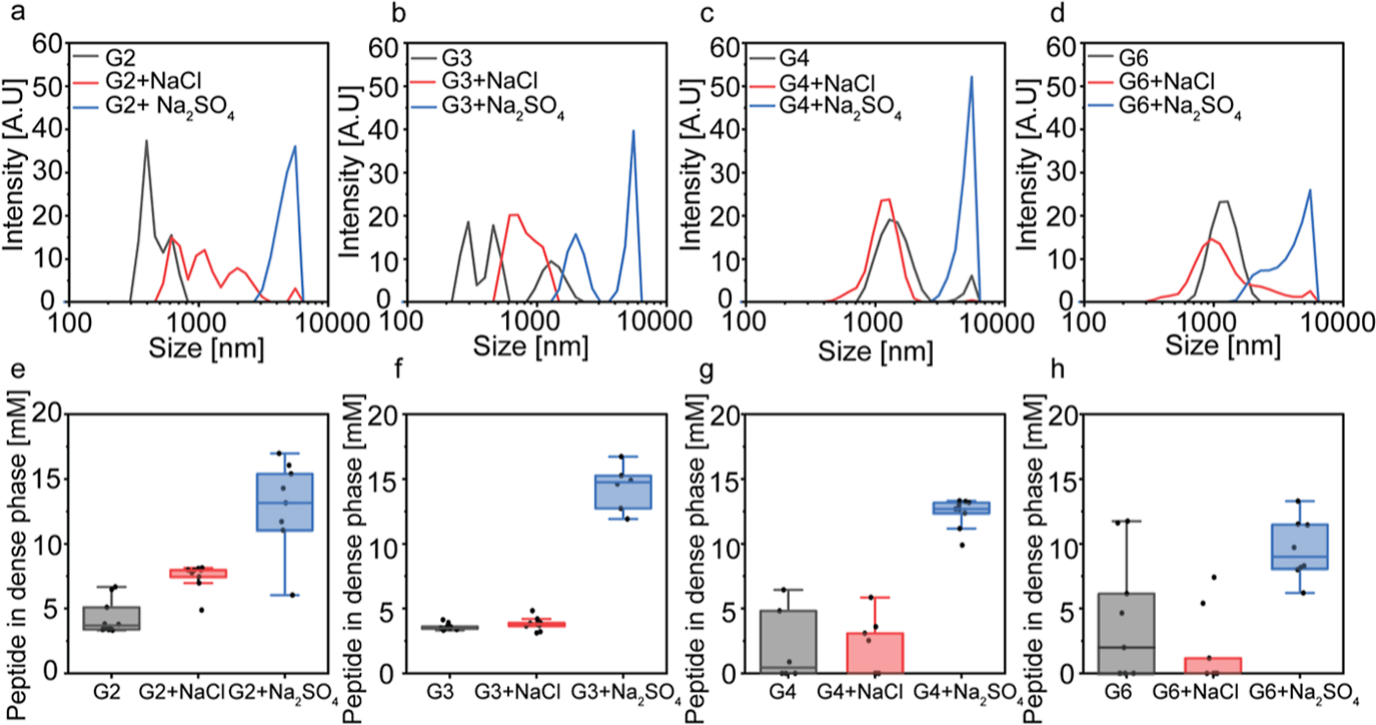
Divalent ions increase the condensate diameter and dense-phase concentration of peptides. (a–d) DLS analysis of 20 mM G2 (a), G3 (b) G4 (c), and G6 (d) condensates in phosphate buffer, pH 7.5, in the absence or the presence of 20 mM NaCl or Na_2_SO_4_. Values represent averages of three independent repeats with 10–12 scans each. (e–h) Peptide concentration in the dense phase of G2 (d), G3 (f), G4 (e), and G6 (f), in the absence or the presence of 20 mM NaCl or Na_2_SO_4_. The total peptide concentration is 20 mM in each system. Quantification of peptide concentration was performed by absorbance spectroscopy using calibration curves for each peptide. Boxplots represent three independent analyses. (a–d) Average data points of *n* = 3, (e–h) show *n* = 9 for 3 independent experiments.

Next, to shed light on how the glycine content and salt composition affect phase behavior, we analyzed the concentration of the peptides in the dilute phase for each system. For this, LLPS samples were centrifuged, and the supernatants (dilute phase) were collected and analyzed by absorbance spectroscopy based on tryptophan absorbance (λ = 280 nm), using calibration curves for each peptide (Figure S7). We found that similar to the trend observed by the microscopy analysis (Figure 2), the concentrations of G2 and G3 in the dense phase in the presence of NaCl (7.5 ± 1.0 and 3.8 ± 0.5 mM, respectively) (Figure 3e–h and Table S1) are 4-fold and 2-fold higher than those of G4 (1.7 ± 2.2 mM) and G6 (1.6 ± 1.9 mM), respectively. In Na_2_SO_4_, the dense-phase concentration for all peptides increases markedly to 12.8 ± 3.4, 10.7 ± 5.2, 12.4 ± 1.2, and 10.7 ± 4.3 mM for G2, G3, G4, and G6, respectively. The higher peptide concentration in the dense phase in the presence of Na_2_SO_4_ can explain the increase in condensate diameter in the presence of the divalent anion observed by DLS analysis (Figure 3a–d). Together, the phase diagram analysis (Figure 2), DLS, and the quantitative analyses of dense-phase peptide concentration (Figure 3) suggest that the shorter and more rigid, glycine-poor peptides G2 and G3 have higher phase separation propensity in NaCl and that the kosmotrope sulfate anion (SO_4_^2–^) increases LLPS propensity for all peptides. The sulfate anion likely acts through two complementary mechanisms to promote condensate formation by more effectively screening interpeptide electrostatic repulsion than monovalent ions and by mediating water-structuring effects that favor closer peptide–peptide contacts. Consistent with this, our dilute-phase measurements show that G2 exhibits the lowest peptide concentration in the supernatant and therefore the highest peptide packing in the dense phase, under the conditions tested. We propose that the reduced backbone flexibility of the glycine-poor G2 sequence promotes stronger, more persistent intermolecular attractions and concomitantly reduces peptide solvation. This combination of stronger attractive forces and reduced hydration would rationally explain the higher peptide density observed in the dense phase for G2. To complement this analysis, we analyzed the water content^42^ in the dense phase of the various peptide condensates formed with NaCl vs Na_2_SO_4_. The results show that salt identity has a pronounced influence on dense-phase hydration, while differences between the peptides were minimal. Relative to NaCl, Na_2_SO_4_ reduced the water content of the dense phase by 38%, 40%, 25%, and 46% for G2, G3, G4, and G6, respectively (Figure S8). Sulfate ions are strong kosmotropes compared to chlorides, and thus, they likely promote LLPS in different modes. Kosmotropic sulfate ions bind strongly to water molecules, decreasing the hydration of amino acid side chains and thereby promoting intermolecular interactions. The water content analysis results suggest that kosmotropic sulfate enhances dense-phase packing by reducing the water content, thereby increasing the local peptide concentration and strengthening LLPS.

To gain more insight into the adopted peptide conformation and H-bonding network, we performed circular dichroism (CD) and attenuated total reflectance-Fourier transform infrared (ATR-FTIR) analyses. CD spectra were similar across all peptides and in the absence or presence of NaCl or Na_2_SO_4_, showing a pronounced negative minimum at ∼192 nm and two positive maxima at 200 and 225 nm, consistent with predominant random-coil conformations (Figure S9a–d). The ATR-FTIR analysis revealed amide I bands at 1640–1650 cm^–1^ for G3, G4, and G6, with no detectable differences between salt-free and Na_2_SO_4_ conditions (Figure S9f–h). In contrast, G2 exhibited a shift from ∼1640 cm^–1^ to ∼1677 cm^–1^ in the presence of Na_2_SO_4_ (Figure S9e), indicative of salt-induced order-to-disorder shift unique to this sequence. The distinct behavior of G2 might be a result of its higher charge and aromatic density and its shorter and more rigid backbone, which may promote phase separation, as shown by the phase diagram analysis (Figure 2a).

### Higher Glycine Content Increases Peptide Chain Flexibility and Condensate Diffusivity

To learn how the glycine content affects the material properties of condensates, we performed fluorescence recovery after photobleaching (FRAP) analysis using confocal laser scanning microscopy (CLSM) for each peptide in the presence of Na_2_SO_4_. For this, we used rhodamine B (10 μM) as a fluorescent payload and a probe and monitored the recovery of its fluorescent signal within the condensates after photobleaching (Figure 4a). We first confirmed that the encapsulation efficiency (EE) of the dye is similar across the various peptide condensates. For this, we quantified the concentration of rhodamine B in the dilute phase of each system following centrifugation, using absorbance spectroscopy (λ = 560 nm) and based on an appropriate calibration curve (Figure S10a). The analysis showed that all peptide condensates have EE of >95% (Figure S10b). Based on the FRAP analysis, the glycine content produces a pronounced and monotonic effect on internal mobility (Figure 4a). G6 condensates display full recovery with a fast *t*_1/2_ (5.1 ± 0.4 s), G4 shows intermediate recovery and slower kinetics (*t*_1/2_ = 15.6 ± 1.2 s), and G3 (*t*_1/2_ = 34.9 ± 1.9 s) and G2 (*t*_1/2_ = 33.3 ± 1.8 s) exhibited the slowest and most incomplete recovery (∼70%) (Figure 4b,c). These results indicate that a higher glycine content increases chain flexibility and internal fluidity, while glycine-poor sequences form more viscous or partially arrested dense phases. Importantly, the slower diffusion in G3 and G2 is consistent with their ability to form denser, possibly more hydrophobic, condensates with higher dense-phase concentration (Figure 3e,f) and may explain their comparatively lower encapsulation of some payloads (see below) (Figure 5).

**Figure 4:**
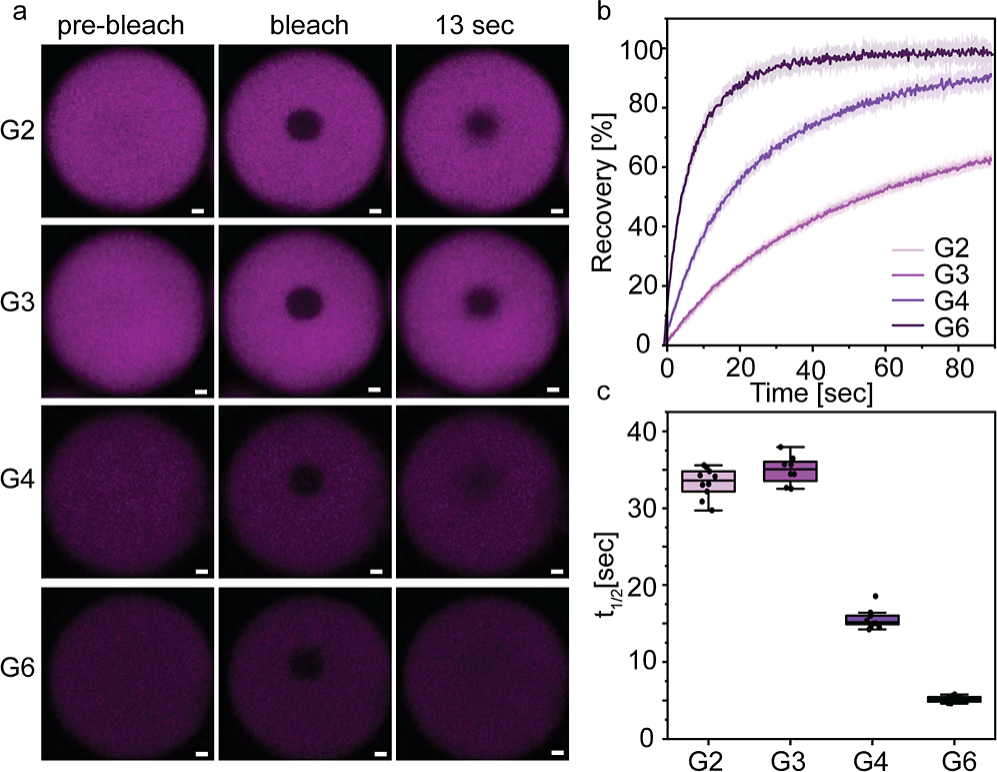
Diffusivity of the condensate is affected by the number of glycine residues. CLSM FRAP analysis using rhodamine B (10 μM) as a fluorescent payload (λ_ex_ = 561 nm). (a) CLSM images of G2, G3, G4, and G6 condensates before, immediately after, and 13 s after rhodamine B photobleaching. Condensates were formed at 20 mM of each peptide in phosphate buffer pH 7.5 with 20 mM Na_2_SO_4_. (b,c) Recovery plots (b) and the respective *t*_1/2_ values (c) obtained from the FRAP analysis. The curves of G2 and G3 are overlapping. Values represent averages (panel b) or boxplots (panel c) of *n* = 10 condensates. Scale bar = 2 μm.

**Figure 5:**
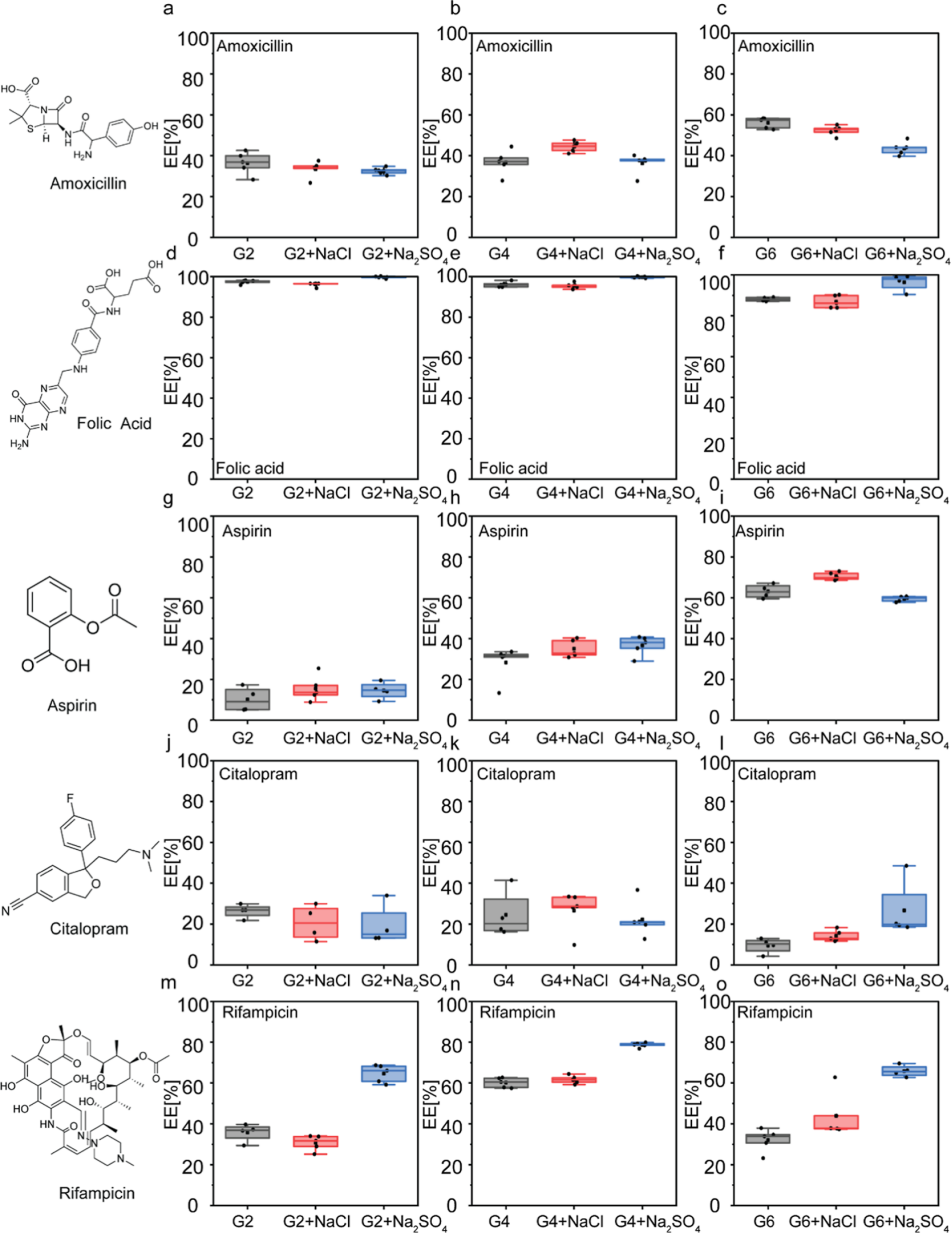
Glycine and salt composition affect the recruitment of therapeutic compounds to condensates. EE of amoxicillin (a–c), folic acid (d–f), aspirin (g–i), citalopram (j–l), and rifampicin (m–o) in condensates formed by 20 mM G2 (a,d,g,j), G4 (b,e,h,k,n), and G6 (c,f,i,l,o) in phosphate buffer pH 7.5 in the absence or presence of 20 mM NaCl or Na_2_SO_4_. Chemical structures of the compounds are presented on the left panel. EE was analyzed by high-performance liquid chromatography (HPLC) using calibration curves for each compound. Boxplots represent 4–5 independent repeats.

### Encapsulation of Therapeutic Compounds in Peptide Condensates Depends on Ion Composition

Owing to the immense potential of peptide condensates as delivery vehicles and the growing number of studies that exploit peptide-based LLPS systems for payload delivery,^14,43–47^ we sought to systematically evaluate how the peptide glycine content and salt identity modulate the recruitment of clinically relevant small-molecule therapeutics. We intentionally selected five FDA-approved compounds that span a wide range of hydrophobicity and ionization behaviors (Table S2): the β-lactam antibiotic amoxicillin (Log *P* = −0.58; p*K*_a_ = 3.2, 6.5, 9.4), the vitamin folic acid (Log *P* = −0.82; p*K*_a_ = 3.5, 4.1), the nonsteroidal anti-inflammatory aspirin (Log *P* = 1.18; p*K*_a_ = 3.3), the selective serotonin reuptake inhibitor citalopram (Log *P* = 3.86; p*K*_a_ = 9.9), and the large, hydrophobic antibiotic rifampicin (Log *P* = 3.85; p*K*_a_ = 5.3, 8.3, 9.3, 11.9, 14.1, 15.9). EE for each compound was determined in condensates formed by the three peptide variants (G2, G4, and G6) in three ionic conditions: no added salt and with 20 mM of NaCl and Na_2_SO_4_. For each condition, samples were centrifuged to separate the dilute and dense phases, and the supernatants were quantified by HPLC against compound-specific calibration curves (Figure S11).

A clear structure–property relationship emerged regarding how drug polarity, hydrophobicity, and charge influence EE. Overall, EE is primarily dictated by drug polarity and charge, with only minor differences observed among the peptide systems (Figure 4 and Tables S3–S5). Polar and negatively charged drugs exhibit the highest EE. For example, folic acid (Log *P* = −0.82; charge = −2) shows consistently high EE across all peptides, likely due to favorable electrostatic interactions with the cationic R side chains. In contrast, increasing hydrophobicity reduced EE, as seen for the negatively charged but relatively hydrophobic aspirin (Log *P* = 1.18, charge = −1), and even more so for the hydrophobic and positively charged citalopram (Log *P* = 3.86, charge = +1), whose low EE is likely driven by electrostatic repulsion from the R side chains within the peptides. Interestingly, rifampicin (Log *P* = 3.85, charge = −1) maintains a relatively high EE despite its hydrophobicity, which we attribute to its multiple hydroxyl groups that may promote hydrogen bonding with the peptide.

Importantly, while differences among peptide sequences were minimal, we observed a strong dependence on salt composition, as introducing Na_2_SO_4_ increased the EE of rifampicin by 28% (G2), 18% (G4), and 34% (G6) relative to the salt-free condition. This is consistent with the higher dense-phase peptide content promoted by the kosmotrope sulfate ions (Figure 3e–h). We therefore attribute this enhancement primarily to increased dense-phase peptide packing and reduced water content, which together create a more favorable hydrophobic microenvironment and promote partitioning of hydrophobic compounds such as rifampicin and, to a lesser extent, citalopram. In contrast, the sulfate-induced hydrophobic environment is unlikely to enhance encapsulation of the more polar compounds amoxicillin, folic acid, and aspirin. At the molecular level, effective electrostatic screening strengthens hydrophobic and π-mediated interactions over charge-driven interactions. This observation was previously reported in arginine-rich phase-separated systems^36^ and can also explain the enhanced encapsulation of the apolar rifampicin with Na_2_SO_4_. Taken together, these results indicate that both polarity/hydrophobicity and charge are key determinants of small-molecule recruitment into peptide condensates but that salt identity modulates these baseline tendencies. These findings, supported by FRAP (Figure 4) and dense-phase concentration (Figure 3e–h) analyses, provide design rules for tuning peptide condensates toward selective sequestration of small-molecule therapeutics.

### Peptide Condensate Disassembly Following Enzymatic Proteolysis

Lastly, we sought to further examine the capacity of the condensates to serve as a delivery platform by probing stimulus-triggered disassembly under a physiologically relevant proteolytic challenge using trypsin as a model protease. We first examined the localization and distribution of fluorescently labeled trypsin within the condensates. For this, we labeled trypsin with Atto 633 and analyzed its fluorescence intensity in the condensates at *t* = 0 and 10 min after enzyme addition. The analysis showed that enzyme diffusion is slower in larger droplets than in smaller ones (Figure S12). In small-diameter condensates, the enzyme becomes homogeneously distributed, whereas in large droplets, it is predominantly localized at the outer shell at *t* = 10 min, due to the slower kinetics.

To analyze and quantify peptide degradation in the LLPS systems following proteolysis, we performed an HPLC analysis of trypsin-treated condensates at increasing time points. Following trypsin addition to the condensates, the reactions were quenched at defined time points (1, 10, 20, 30, 40, 50, and 60 min) by the addition of acetonitrile (ACN) with 1% trifluoroacetic acid (TFA). The entire condensate reaction mixtures were then injected into the HPLC system, and the intact peptide concentration was quantified using peptide-specific calibration curves (Figure S13). After 60 min of trypsin treatment, the concentrations of the intact peptide were 10.0 ± 3.5 mM for G2, 5.9 ± 1.2 mM for G4, and 9.5 ± 0.6 mM for G6 (Figure 6a–c). These results indicate that G4 undergoes the most extensive hydrolysis, whereas G2 and G6 exhibit comparable levels of degradation. In addition, we performed time-lapsed CLSM analysis to monitor trypsin-induced degradation of condensates loaded with rifampicin, chosen as a model payload due to its intrinsic fluorescence (λ_ex_ = 475 nm). Preformed rifampicin-loaded condensates (450 μM rifampicin; 20 mM peptide) were incubated in phosphate buffer containing 20 mM Na_2_SO_4_, after which trypsin (500 nM) was introduced to trigger enzymatic disassembly. Upon trypsin addition, all three peptide systems exhibited a time-dependent decrease in condensate abundance and diameter (Figure 6d,f,h) compared to untreated condensates (Figure S14). G4 condensates displayed the most pronounced and rapid disassembly; within 30 min, droplets lost their spherical morphology and underwent near-complete disassembly (Figure 6e). G6 condensates showed an intermediate behavior, with progressive shrinkage and partial loss of integrity over 60 min, where droplets transitioned into irregular, nonspherical aggregates rather than dissolving into a homogeneous dilute phase. By contrast, G2 condensates underwent only moderate apparent disassembly (Figure 6d). This trend is in strong agreement with the HPLC analysis which showed the highest degree of peptide hydrolysis for G4. To assess the rifampicin release from peptide condensates upon enzymatic proteolysis, we quantified dilute-phase rifampicin by HPLC at *t* = 30 min for trypsin-treated and untreated droplets. The percentage of release was calculated as the dilute-phase concentration at *t* = 30 min relative to the total rifampicin concentration (see Experimental Section). Overall, G6 condensates exhibited the highest rifampicin release (87.4 ± 6.1%), while G2 and G4 condensates showed comparable release efficiencies (79.8 ± 1.0% and 74.2 ± 4.7%, respectively; Figure 6e,g,i). Notably, proteolytic degradation had the strongest impact on rifampicin release from G2 condensates (Figure 6e), with a >20% increase upon trypsin treatment, compared to ∼18% and ∼11% increases for G4 and G6 condensates, respectively.

**Figure 6:**
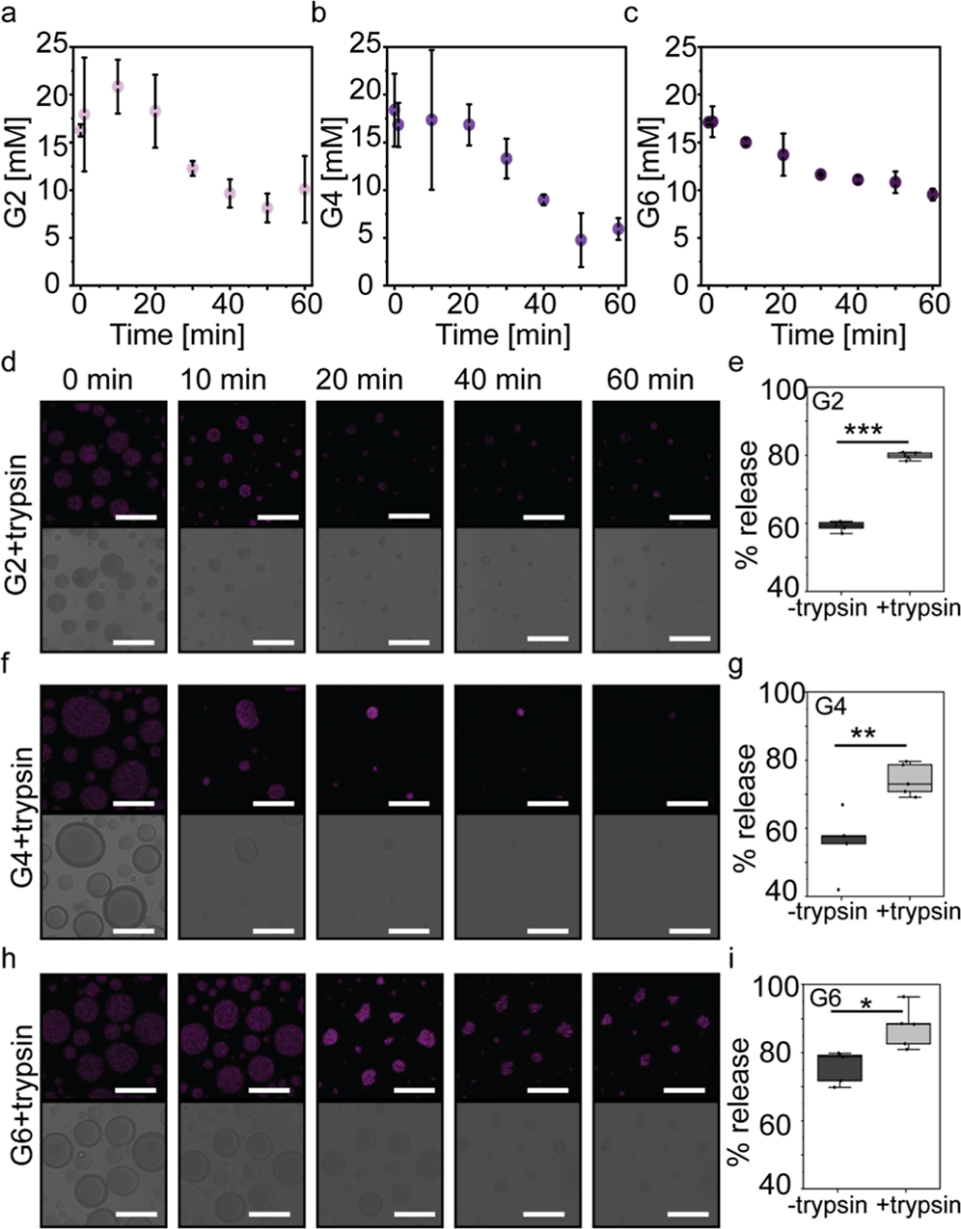
Condensate disassembly following enzymatic proteolysis. (a–c) HPLC analysis of peptide concentration in condensates formed by G2 (a), G4 (b), and G6 (c) following proteolytic hydrolysis at increasing time points. (d,f,h) CLSM analysis of preformed condensates (20 mM peptide in phosphate buffer pH 7.5 with 20 mM Na_2_SO_4_) loaded with the antimicrobial compound rifampicin (450 μM) and subsequently treated with trypsin (500 nM). Scale bar = 20 μm. (e,g,i) HPLC analysis of rifampicin release (%) from untreated condensates and condensates treated with trypsin (500 nM), both at *t* = 30 min. % release was calculated based on the dilute-phase rifampicin concentration after centrifugation and using an HPLC standard curve. Boxplots represent 3 independent analyses. *p-value <0.05, **p-value <0.01, and ***p-value <0.001.

Together, these findings demonstrate that the glycine content, which modulates charge and aromatic density and backbone flexibility, governs not only static partitioning and internal dynamics but also the susceptibility of peptide condensates to enzymatic activation. An intermediate glycine composition (G4) produces a favorable combination of payload loading and efficient protease-mediated disassembly, whereas glycine-poor (G2) condensates are more resistant to cleavage, and glycine-rich (G6) condensates show only partial responsiveness. Nonetheless, for translational drug delivery applications, additional considerations remain critical, particularly stability in physiologically relevant environments. For example, the presence of serum (10%) in cell culture medium markedly altered the condensate properties (Figure S15). As such, ongoing efforts in the field are increasingly focused on designing synthetic condensates that maintain functionality within complex biological environments.

## Conclusions

In summary, systematic studies of glycine content in short cationic and aromatic-rich peptides provide a useful means to tune the properties of peptide condensates that are critical for their capacity to serve as delivery vehicles. Glycine content regulates charge density, aromatic density, and backbone flexibility that govern LLPS propensity, dense-phase peptide concentration, dense phase diffusivity, and in turn, the recruitment of small-molecule therapeutics with varying chemical properties.

Compared to NaCl, Na_2_SO_4_ promotes LLPS more strongly, likely through the dehydration of peptide side chains due to its stronger binding affinity to water molecules. As a strong kosmotrope anion, sulfate enhances dense-phase packing and improves the encapsulation of apolar compounds by increasing the local peptide concentration, decreasing the water content, and strengthening the hydrophobic and π-mediated interactions.^36^ Importantly, the glycine composition also dictates protease responsiveness and the kinetics of disassembly. Intermediate glycine number provides a balance between efficient payload encapsulation and rapid protease-triggered disassembly, whereas glycine-poor sequences form highly packed and proteolytically resistant condensates. These insights can be further implemented for engineering peptide-based condensates as delivery vehicles for small-molecule therapeutics. For instance, selection of peptide sequences, e.g., G2, promotes the formation of slowly diffusing condensates with a slower disassembly for slow-release applications, or sequences that allow triggered payload release in protease-rich microenvironments, e.g., G4. Future work should extend these findings to physiologically relevant proteases, explore responsiveness to additional stimuli, and investigate in vivo stability. Collectively, our results facilitate the rational design of peptide condensates as tunable microcompartments for selective sequestration and controlled release of therapeutics.

## Experimental Section

### Materials and Reagents

Peptides were custom-synthesized and purified by GenScript (Hong Kong). Dimethyl sulfoxide (DMSO), rhodamine B, and sodium sulfate (Na_2_SO_4_) were obtained from ACROS Organics. Folic acid and citalopram were purchased from Aaron Chemicals. Acetylsalicylic acid (aspirin), RPMI-1640 medium, Atto 633 protein-labeling kit, and trypsin were obtained from Sigma-Aldrich. Amoxicillin, rifampin, sodium phosphate monobasic, and sodium phosphate dibasic were purchased from Thermo Scientific. Sodium chloride (NaCl), sodium hydroxide (NaOH), and hydrochloric acid (HCl) were obtained from Biolab.

### Phase Diagrams

Peptide stock solutions were prepared in 20 mM phosphate buffer (pH 7.5) or in 20 mM Tris buffer pH 7.5 (for the analysis presented in Figure S5) at final concentrations of 2.5, 5, 10, and 20 mM. These solutions were mixed with 10% (v/v) NaCl or Na_2_SO_4_ prepared in ddw (solution B), yielding final salt concentrations of 5, 10, 20, and 50 mM. For Na_2_SO_4_, additional concentrations of 1 and 2.5 mM were also tested. Condensates were formed immediately upon mixing. All measurements were conducted at room temperature. Bright-field images were acquired at the indicated concentrations using a fluorescence microscope (Olympus IX83) equipped with a 40×/0.95 NA Universal Plan Extended Apochromat objective. Images were collected and processed by using CellSens Dimension software. Turbidity was measured in triplicates by optical density at λ = 350 nm in a BioTek H1 synergy plate reader using a 384-well plate. To analyze the distribution of diameters of the condensates, we used the software ImageJ.

### Peptide Condensate Solution Preparation

Peptides were dissolved in 20 mM phosphate buffer (pH 7.5) at a final concentration of 20 mM, and the pH was adjusted to 7.5. Either NaCl or Na_2_SO_4_ was then added from a 200 mM stock solution to a final concentration of 20 mM. Condensates were formed upon pH adjustment. For samples prepared without salt, ddw was added instead.

### DLS Analysis

Condensates were prepared as described above and transferred to disposable ZEN0040 cuvettes. Measurements were performed using a Zetasizer Nano Z (Malvern Instruments). Each system was analyzed three independent times, with 10–12 scans recorded per measurement.

### Quantification of Peptide Concentration in the Dilute Phase

Condensates were prepared as described above. Droplet suspensions were centrifuged at 20 °C and 17k rcf for 45 min. The supernatants were collected and analyzed by absorbance spectroscopy at λ = 280 nm using a Synergy H1 plate reader (BioTek).

For quantification, nine independent calibration curves were generated (three for each peptide). Peptides were dissolved in phosphate buffer (20 mM, pH 7.5) containing 20 mM salt (NaCl, Na_2_SO_4_, or ddw to maintain dilution) and serially diluted to final concentrations of 0.02, 0.05, 0.1, 0.2, 0.4, 0.6, and 0.8 mM. Supernatants were further diluted (1:20) in phosphate buffer with the corresponding salt, and absorbance was recorded in triplicate at 280 nm. Peptide concentrations in the dilute phase were determined by using the corresponding calibration curves.

### Water Content Measurement

Condensates solutions were prepared as described above at a volume of 500 μL in preweighted Eppendorfs. Then, the solutions were centrifuged at 20 °C at 25,830 rcf for 1 h. After centrifugation, the supernatant was extracted, and the pellets were weighed. After weighing the pellets, they were lyophilized overnight and were weighed again. The water content was calculated as

1 $$water\;content=\frac{m_{wet\;pellet}-m_{dry\;pellet}}{m_{wet\;pellet}}\times 100\%$$

where *m*_wet pellet_ is the mass of the pellet before lyophilization, and *m*_dry pellet_ is the mass of the pellet after lyophilization. For peptide G3, only a single repeat for each salt was made at a condensate solution volume of 300 μL.

### CD Spectroscopy (CD) Analysis

CD spectroscopy experiments were performed at room temperature between 190 and 260 nm, at a bandwidth of 1 nm and scanning speed of 0.5 s per point, using a Chirascan V100 CD instrument. Samples were prepared as previously described. To achieve an appropriate absorption, all samples were diluted 1:20 in ddw, and a mountable cuvette with a path length of 0.1 mm was used to suit the final concentration of 1 mM. An average of four scan analyses was recorded for each sample.

### ATR-FTIR Analysis

Samples were prepared as previously described. FTIR analysis was performed on dry peptide powders or lyophilized samples of peptide droplets with Na_2_SO_4_. The samples were recorded using ATR with a diamond crystal, using Bruker Tensor 27 IR with a clean crystal recorded as blank for each sample. Absorbance spectra were measured within the range 0–4000 cm^–1^. The presented spectra show an average of 4 repeats.

### FRAP Analysis

FRAP experiments were performed by using a Zeiss LSM 900 confocal microscope to track the fluorescence of 10 μM rhodamine B as a payload. Condensates were formed by mixing 20 mM of each peptide with 20 mM Na_2_SO_4_ in 20 mM phosphate buffer (pH 7.5). All solutions were imaged in Pluronic F-127-coated 96-well plates (5 mg/mL in buffer) according to a previously reported protocol by Yao and Rosen.^48^ Photobleaching was performed on a circular region of interest (ROI) with a diameter of 2.5 μm with a reference ROI of the same size and a background reference ROI of 1 μm. Ten iterations of the 561 nm excitation laser at 100% intensity were applied for rhodamine B using a 40×/1.2 Imm Korr DIC M27 objective. Fluorescence recovery at the bleached area was recorded and analyzed using Zen Blue 3.2 software. Photobleaching correction and recovery times were calculated using OriginLab version 9.95. Intensity data were normalized between 0% (bleach intensity) and 100% (prebleach intensity) using the following equations:

2 $$I_{corrected}\left(t\right)=R_{1}\left(t\right)-R_{3}\left(t\right)\times \frac{{R_{2}}^{pre}-{R_{2}}^{pre}}{R_{2}\left(t\right)-R_{3}\left(t\right)}$$

for raw data correction, where *R*_1_(*t*) is the FRAP region at time *t*, *R*_2_(*t*) is the reference region at time *t*, *R*_3_(*t*) is the background reference, *R*_2_^pre^ is the prebleach average for the reference area (*R*_2_), and *R*_3_^pre^ is the prebleach average for the background area (*R*_3_):

3 $$I_{normalized}\left(t\right)=\left(\frac{I_{corrected}\left(t\right)-I_{bleach}}{I_{pre}-I_{bleach}}\right)\times 100$$

for min–max scaling (0–100% normalization), where: *I*_corrected_(*t*) is the calculated intensity from eq 2, *I*_bleach_ is intensity at the bleach time at the bleach area (*R*_1_), and *I*_pre_ is the average intensity of *R*_1_ before bleaching. Then, the normalized data were fitted to the next equation to extract the fitting parameters:

4 $$y=a-bc^{x}$$

where *a* is the recovery that was measured. *t*_1/2_ values of WGI-0 and WGI-1 were calculated for each experiment using *a*, *b*, and *c* extracted from the fitting, with the following equation:

5 $${t_{1/2}=\log}_{c}\left(\frac{0.5\times a}{b}\right)$$

The final FRAP recovery curves and the *t*_1/2_ values represent averages of the recovery plots collected from *n* = 8–10 condensates for each sample.

### Encapsulation Efficiency Analysis of Rhodamine B Using Absorbance Spectroscopy

Condensates were formed by dissolving each peptide at 20 mM in phosphate buffer (pH, 7.5) and then adding rhodamine B (10 μM, dissolved in ddw). Subsequently, 200 mM NaSO_4_ was added (final concentration of 20 mM). Droplet solutions were centrifuged at 20 °C at 17k rcf for 45 min. Supernatants were collected from each sample and analyzed using absorbance spectroscopy at λ = 560 nm using a BioTek H1 synergy plate reader. To obtain the concentrations of rhodamine B in the supernatants, we used a calibration curve. For this, rhodamine B was dissolved in ddw in a stock of 1 mM, then diluted in buffer to a final concentration of 10 μM, and serially diluted to final concentrations: 0.5, 1, 1.5, 2, 4, 6, 8 μM. The absorbance of each sample was measured in triplicates at λ = 560 nm. The %EE values were obtained using the following equation:

6 $$\%EE=\left(\frac{\left(C_{i}-C_{\sup}\right)}{C_{i}}\right)\times 100$$

where *C*_i_ represents the initial (total) concentration of rhodamine B, and *C*_sup_ represents the rhodamine B concentration in the supernatant.

### HPLC Encapsulation Efficiency Analysis of Amoxicillin, Folic Acid, Aspirin, Citalopram, and Rifampin

The predicted Log *P* and p*K*_a_ values of the drugs were obtained from ChemDraw. The EE of amoxicillin, folic acid, aspirin, citalopram, and rifampin in peptide condensates was evaluated by HPLC. Compounds were initially dissolved at 50 mM in DMSO. Peptides were dissolved at 20 mM in 20 mM phosphate buffer (pH 7.5) with a subsequent pH adjustment. Subsequently, the stock solution was diluted 1:99 to the peptide solution (final concentration, 500 μM), followed by the addition of the corresponding salt or ddw. Condensates formed upon pH adjustment. Droplet suspensions were centrifuged at 20 °C and 17,000*g* for 45 min. For HPLC analysis of the payload in the dilute phase, 100 μL of the supernatant was diluted with 100 μL of ACN. Samples were analyzed using a Dionex Ultimate 3000 UHPLC system (Thermo Fisher) equipped with a diode-array detector (DAD). Separation was performed on a CS Chromatography MultiHigh C18 column (250 × 4.6 mm^2^, 5 μm, 100 Å) using a gradient of mobile phases: (A) H_2_O with 0.1% TFA and (B) ACN with 0.1% TFA. For all compounds except aspirin, the gradient was 5–95% B over 24 min at 1 mL/min; for aspirin, 30–50% B over 24 min at the same flow rate. Calibration curves were generated for each compound. Stock solutions (50 mM in DMSO) were diluted 1:99 in ddw to 500 μM, followed by serial dilutions to 50, 100, 200, 300, and 400 μM. After the addition of 100 μL of ACN, solutions were analyzed under the same HPLC conditions as described above. Peak areas were monitored at 214 nm, except for rifampin, which was monitored at 475 nm. Retention times were as follows: amoxicillin, 9.99 min; folic acid, 10.79 min; aspirin, 8.47 min; citalopram, 17.37 min; and rifampin, 21.62 min. EE (%EE) was calculated using eq 6.

Loading % for each compound was calculated using eq 7:

7 $$loading\;\%=\frac{EE\%\times C_{drug}}{C_{pep}}$$

where EE is the calculated encapsulation efficiency shown in Figure 5, *C*_drug_ is the total molar concentration of each compound (0.5 mM), and *C*_pep_ is the calculated peptide concentration in the dense phase, determined from the dilute-phase measurements shown in Figure 3 and Table S1.

### Trypsin Labeling and CLSM Analysis of Localization and Distribution

Trypsin was labeled using an Atto 633 protein-labeling kit (Sigma). The labeled enzyme was purified using a gel filtration column (included in the kit). The concentration of the labeled enzyme was measured by absorbance at λ = 630 nm and 280 nm using an Agilent Technologies Cary 100 UV–vis spectrophotometer and calculated to be 6 μM using the equation:

8 $$C_{protein}\;\left[\mu M\right]=\frac{A_{280}-\left(0.06\times A_{630}\right)}{\varepsilon_{protein}}\times 10^{6}$$

where *A*_280_ is the absorbance at 280 nm and *A*_630_ is the absorbance at 630 nm. After labeling, Atto 633-trypsin was diluted to a concentration of 5 μM.

Stock solutions were prepared as follows: peptides at 25 mM, trypsin at 5 μM, and Na_2_SO_4_ at 200 mM in ddw, all in 20 mM phosphate buffer (pH 7.5). Condensate solutions were prepared by mixing 22.22 mM peptide and 22.22 mM Na_2_SO_4_. From these solutions, 45 μL was transferred to Pluronic F-127-coated wells (as described by Yao and Rosen), followed by the addition of 5 μL of Atto 633-trypsin solution, yielding final concentrations of 20 mM peptide, 20 mM Na_2_SO_4_, and 500 nM Atto 633-trypsin. The solution was imaged using a Zeiss LSM 900 inverted confocal microscope, using an λ_ex_ = 640 nm laser, and a collection emission range of λ_em_ = 645–700 nm, and objective 40*×*/1.2 Imm Korr DIC M27. Images were taken over time, for 10 min. The collected images were taken using *z*-stack mode. Condensates of each sample were analyzed using the Zen blue 3.2 software (Zeiss) to show the distribution of Atto 633-trypsin in condensates over time, by using line scan analysis.

### HPLC Analysis of Peptide Cleavage by Trypsin

The cleavage of the peptides with trypsin was evaluated by HPLC. Peptides were dissolved in phosphate buffer at a concentration of 22.2 mM pH 7.5; then, Na_2_SO_4_ stock solution of 222 mM was added (final concentration of 22.2 mM), and condensates formed upon pH adjustment. Then, trypsin was added from a stock solution of 5 μM to a final concentration of 500 nm. To analyze reaction kinetics in HPLC, ACN with 1% TFA was added at a final volume percentage of 50% v/v to quench the reaction at the desired time point (0–60 min). Peptide concentration was monitored over time using an analytical reverse-phase HPLC system (Thermo Fisher), a Dionex SD Ultimate 3000 UHPLC standard system equipped with a DAD detector. Mobile phases were (A) H_2_O (0.1% TFA) and (B) ACN (0.1% TFA), and the stationary phase was a CS chromatography MultoHigh C18 column (250 mm × 4.6 mm, 5 μm particle size and 100 Å pore size, 5861272), using gradient: 5–95% mobile phase B for 24 min at a flow rate of 1 mL/min. Time 0 of the reaction represents the peptide that was not treated with trypsin, and buffer was added instead. We obtained peptide concentrations by using a calibration curve of the peptides. For this, the peptides were dissolved at 2 mM stock solution in ddw and then diluted with ddw to 0.1, 0.2, 0.4, 0.6, 0.8, 1 mM. All solutions were diluted once more with ACN + 1% TFA. For peptide quantification, we analyzed the peak area at λ_abs_ = 214 nm as a function of predetermined concentration to create a calibration curve equation using linear regression. Then, we could obtain peptide concentration for each reaction time point based on the calibration curve. Data points represent averages of three independent measurements.

### CLMS Analysis of Rifampicin Release

Rifampicin release from peptide condensates degraded by trypsin was monitored using a Zeiss LSM 900 confocal microscope. Stock solutions were prepared as follows: peptides at 25 mM, trypsin at 5 μM, and Na_2_SO_4_ at 200 mM in ddw, all in 20 mM phosphate buffer (pH 7.5). Rifampicin stock was prepared at 50 mM in DMSO. Condensate solutions were prepared by mixing 22.22 mM peptide, 500 μM rifampicin, and 22.22 mM Na_2_SO_4_. From these solutions, 45 μL was transferred to Pluronic F-127-coated wells (as described by Yao and Rosen), followed by the addition of 5 μL of buffer or trypsin solution, yielding final concentrations of 20 mM peptide, 20 mM Na_2_SO_4_, 450 μM rifampicin, and 500 nM trypsin. The solution was imaged using a Zeiss LSM 900 inverted confocal microscope, using an λ_ex_ = 488 nm laser, and a collection emission range of λ_em_ = 450–700 nm, objective 40*×*/1.2 Imm Korr DIC M27. Images were taken over time, for 60 min. The collected images were taken using *z*-stack mode.

### HPLC Analysis of Rifampicin Release

Rifampicin release was calculated by measuring the concentration in the dilute phase. Peptides were dissolved in phosphate buffer at a concentration of 22.2 mM pH 7.5; then, rifampicin was added (stock solution, 50 mM in DMSO; final concentration, 500 μM), Na_2_SO_4_ stock solution of 222 mM was added (final concentration of 22.2 mM), and finally trypsin (stock solution, 5 μM; final concentration, 500 nM) or buffer as a control was added. The solutions were incubated for 30 min and then centrifuged at 20 °C and 17,000*g* for 45 min. For HPLC analysis of the rifampicin concentration in the dilute phase, 100 μL of supernatant was diluted with 100 μL of ACN and analyzed in the HPLC system as described previously, using the same calibration curve for rifampicin. For the release calculation, the following equation was used:

9 $$\frac{C_{dilute}}{C_{initial}}\times 100\%$$

where *C*_dilute_ is the concentration obtained by HPLC, and *C*_initial_ is the initial concentration of rifampicin.

### Bright-field Microscopy Analysis of Condensates’ Stability in Cell Culture Medium

Peptides were dissolved in cell culture medium (RPMI) containing 10% fetal bovine serum serum at 20 mM. Then, the pH was adjusted to 7.5, and Na_2_SO_4_ was added to a final concentration of 20 mM. Bright-field images were acquired for 2 h using a fluorescence microscope (Olympus IX83) equipped with a 40×/0.95 NA Universal Plan Extended Apochromat objective. Images were collected and processed using CellSens Dimension software.

## Supplementary Material



## Notes

Heat map phase diagrams, HPLC and absorbance spectroscopy calibration curves of compounds and peptides, bright-field microscopy of condensates, CD and ATR-FTIR analyses, size distribution histograms based on microscopy, water content analysis, rhodamine B EE analysis, and CLSM of condensates (PDF)
