# Supplementary material for: Glycine Composition and Ion Valency Tune Phase Behavior and Drug Encapsulation in Designer Peptide Condensates
Source: ACS Appl Mater Interfaces. 2026 Feb 12;18(7):10820–31. doi: 10.1021/acsami.5c19632 (PMC12954654; doi:10.1021/acsami.5c19632)
Supplement: Supplementary file 1 [file am5c19632_si_001.pdf]

Supporting Information

Glycine Composition and Ion Valency Tune Phase Behavior and Drug Encapsulation in Designer Peptide Condensates

Shirel Veretnik[1], Rif Harris[1] and Ayala Lampel[1-4]*

[1]Shmunis School of Biomedicine and Cancer Research, George S. Wise Faculty of Life Sciences, Tel Aviv University, Israel

[2]Center for Nanoscience and Nanotechnology Tel Aviv University, Tel Aviv 69978, Israel

[3]Center for the Physics and Chemistry of Living Systems Tel Aviv University, Tel Aviv 69978, Israel

[4]Leibniz Institute of Polymer Research Dresden Max Bergmann Center of Biomaterials Dresden 01069 Dresden, Germany

**Corresponding author*

Email: ayalalampel@tauex.tau.ac.il

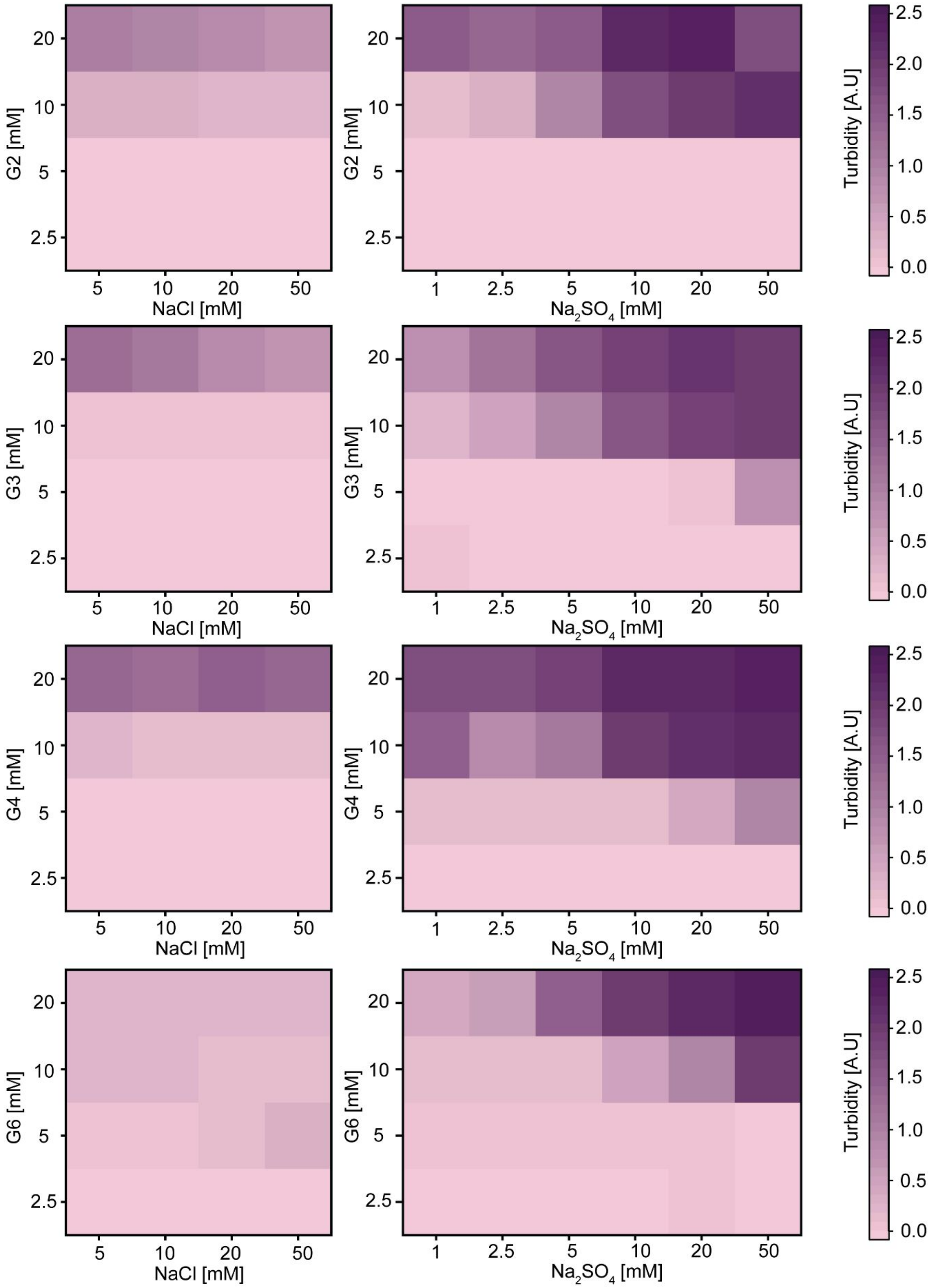

Supplementary Figure 1. Heat map phase diagrams based on turbidity values at 350 nm of G2, G4, and G6 peptides in phosphate buffer pH 7.5 as a function of peptide and salt concentration.

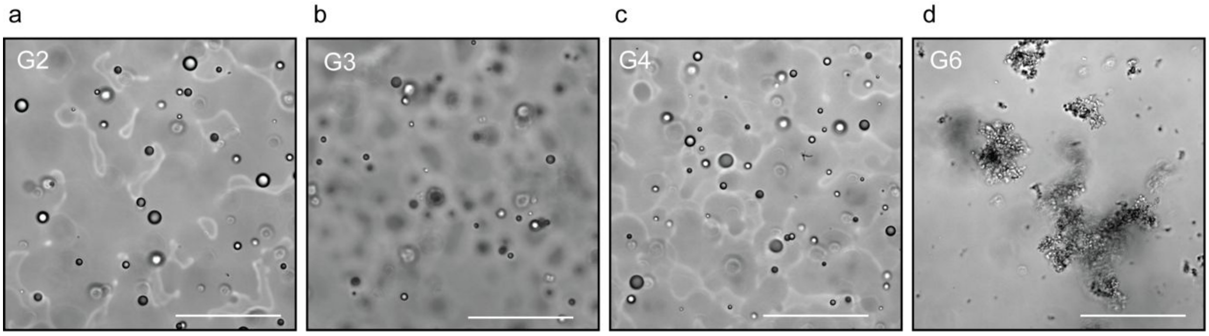

Supplementary Figure 2. Brightfield microscopy analysis of 20 mM of G2 (**a**), G3 (**b**), G4 (**c**) and G6 (**d**) in NaCl and Na_2SO_4-free phosphate buffer pH 7.5. Scale bar=25 μm.

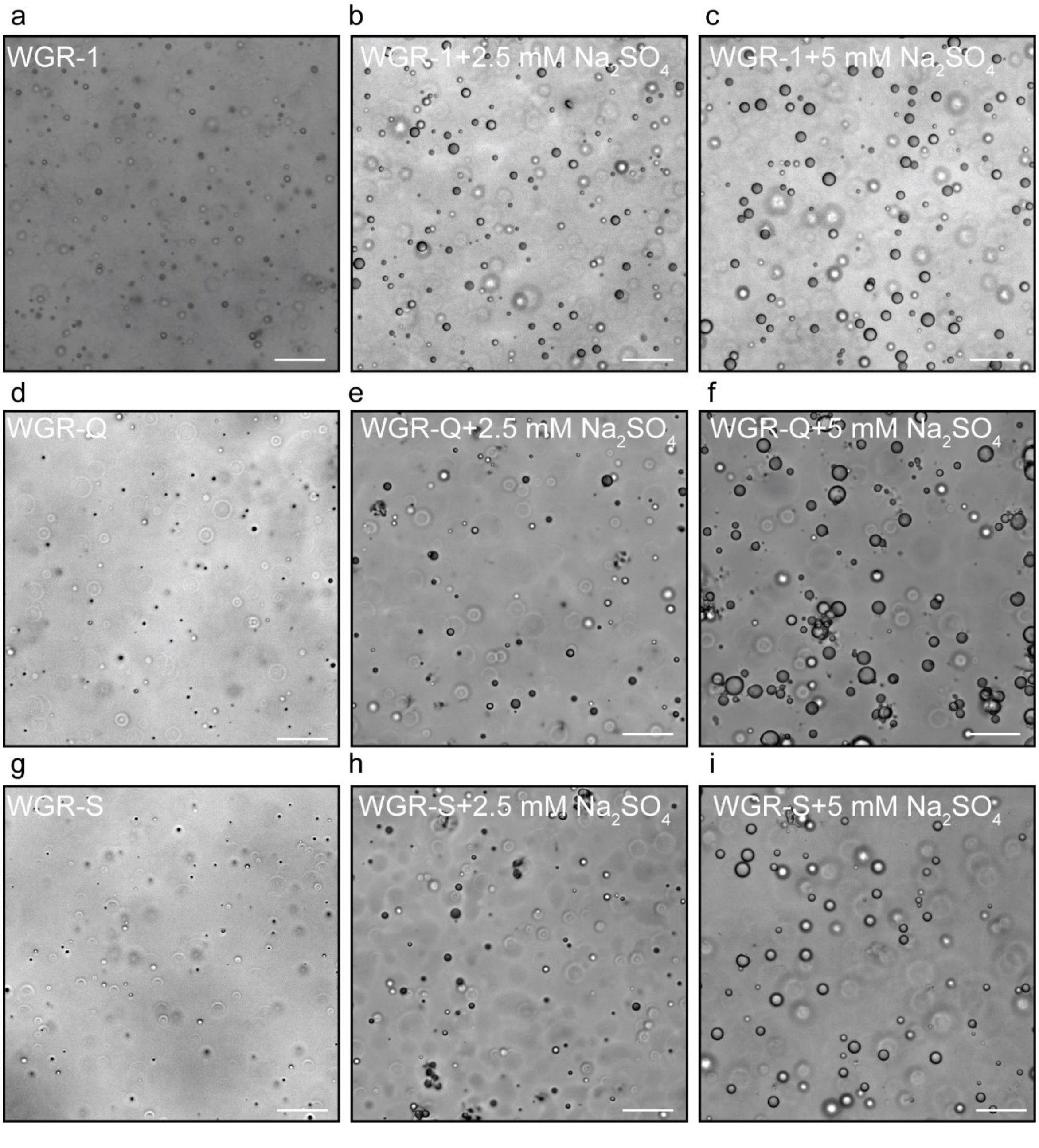

Supplementary Figure 3. Brightfield microscopy analysis of condensates formed by peptides containing six G residues: WGR-1 (WGRGRGRGWPGVGY) (**a-c**), WGR-Q (WGRGRGRGWQGVGY) (**d-f**), and WGR-S (WGRGRGRGWPGSGY) (**g-i**), in the absence (**a, d, g**) or presence of 2.5 mM Na_2SO_4 (**b, e, h**) or 5 mM Na2SO4 (**c, f, i**).Peptide concentration is 20 mM in phosphate buffer pH 7.5. Scale bar=25 μm.

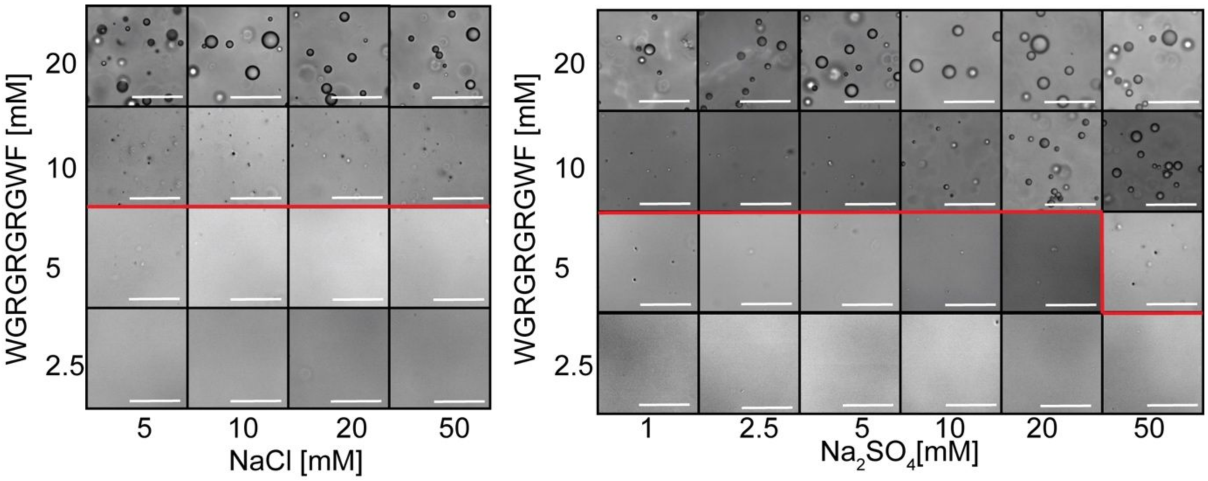

Supplementary Figure 4. Phase diagrams obtained by bright field microscopy analysis of the peptides G4F as a function of peptide, NaCl and Na_2SO_4 concentration. The peptide was dissolved in 2.5, 5, 10 and 20 mM phosphate buffer pH 7.5 and salt was added subsequently. Scale bar=25 μm.

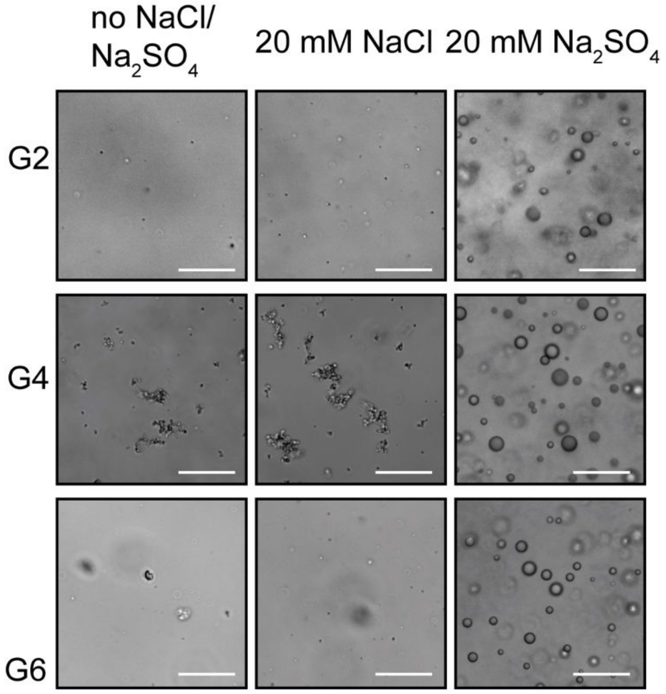

Supplementary Figure 5. Brightfield microscopy analysis of the peptides G2, G4, and G6 (20 mM) in the absence or presence of 20 mM of NaCl and Na_2SO_4. The peptide was dissolved in 20 mM Tris buffer at pH 7.5 and salt or ddw were added subsequently. Scale bar=25 μm.

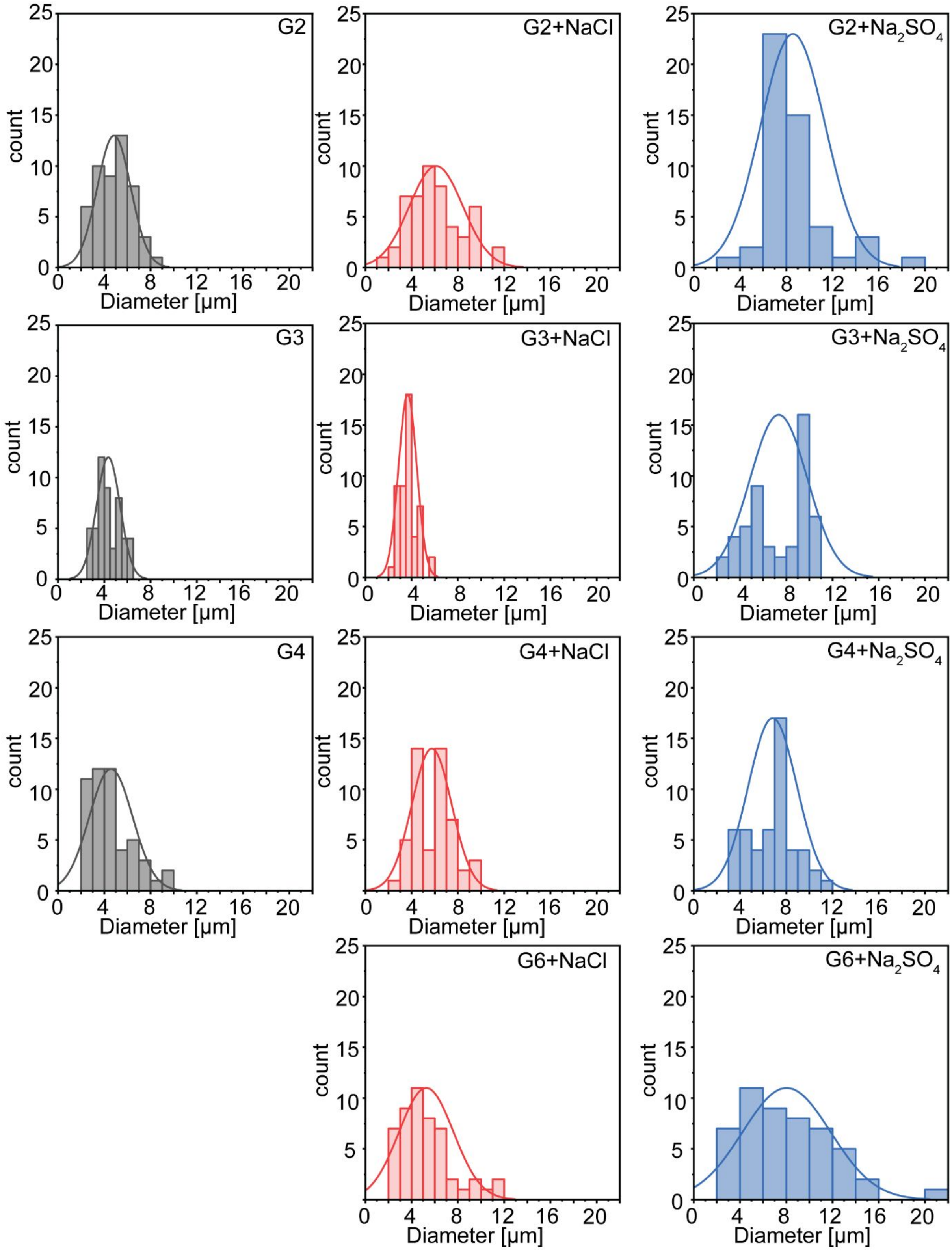

Supplementary Figure 6. Diameter of peptide condensates measured from brightfield microscopy. Histograms showing the diameter of each peptide condensate system (20 mM, in phosphate buffer pH 7.5) in the absence or presence of 20 mM NaCl and Na_2SO_4. Due to the presence of aggregates in G6 without NaCl/Na_2SO_4, we excluded these samples from the analysis. The analysis was performed using ImageJ software.

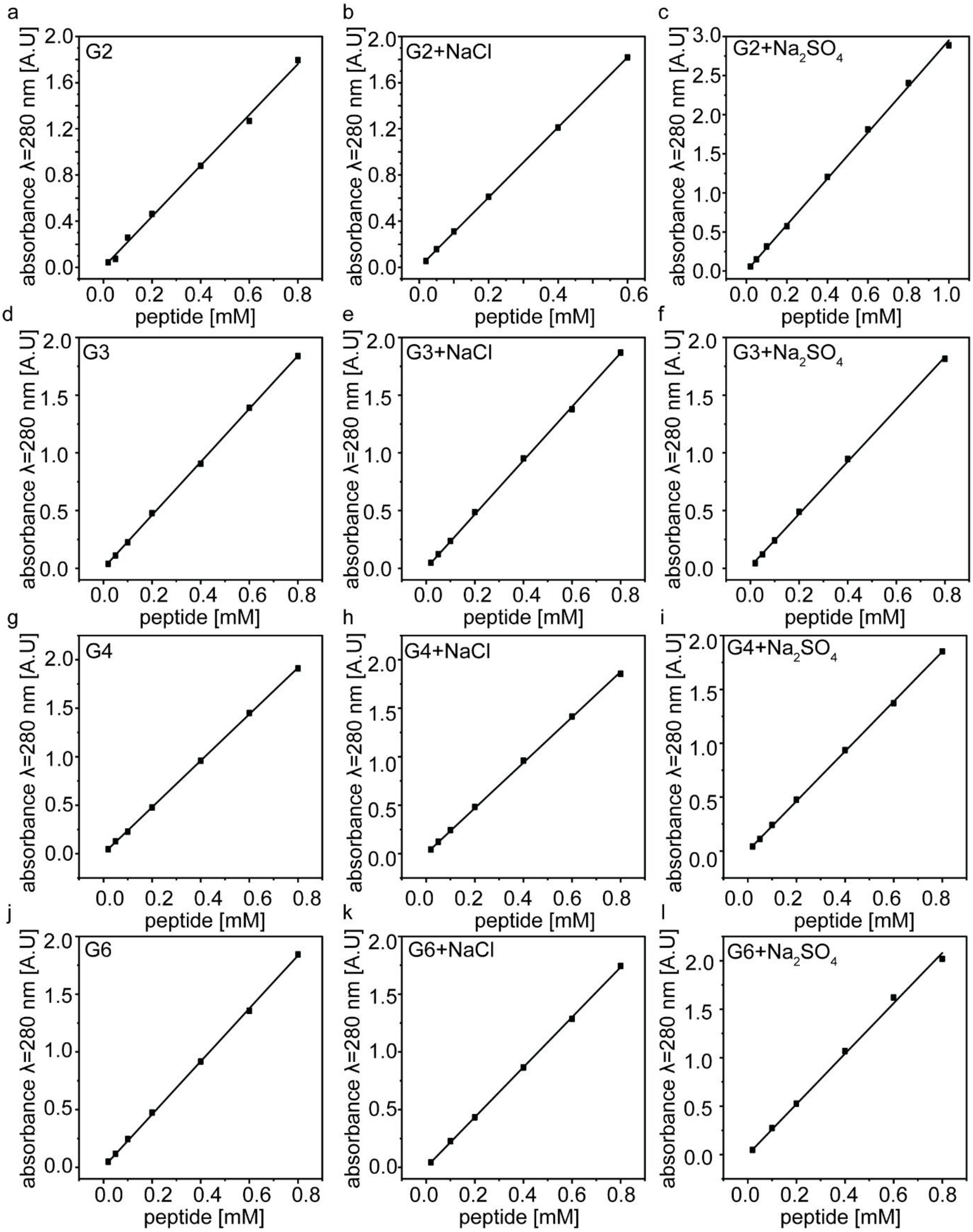

Supplementary Figure 7. Calibration curves of G2 (**a-c**), G3 (**d-f**), G4 (**g-i**) and G6 (j-l) in NaCl and Na_2SO_4-free buffer (a, d, g), or with NaCl (b, e, h) or Na_2SO_4 (c, f, i).

	G2 [mM]	G3 [mM]	G4 [mM]	G6 [mM]
NaCl and Na_2SO_4-free	4.3±1.3	3.6±0.3	2.0±2.9	4.0±4.9
NaCl	7.5±1.0	3.8±0.5	1.7±2.2	1.6±1.9
Na_2SO_4	12.8±3.4	10.7±5.2	12.4±1.2	10.7±4.3

Supplementary Table 1. Peptide concentration [mM] in the dense phase, obtained from supernatants of LLPS samples following centrifugation.

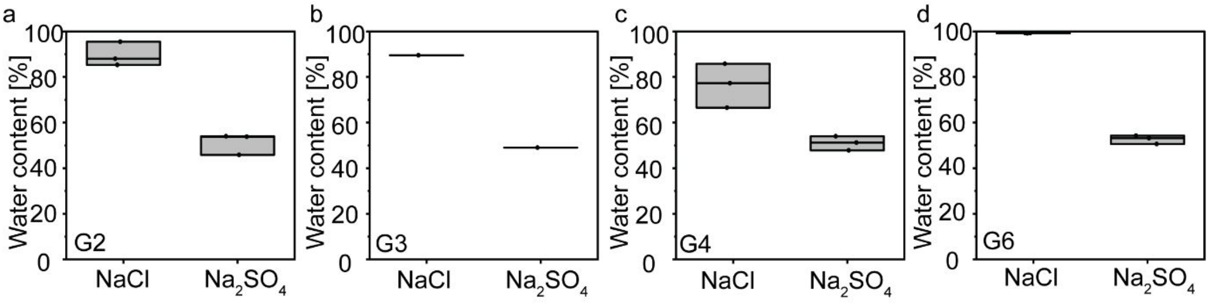

Supplementary Figure 8. Water content analysis of G2 (a), G3 (b), G4 (c), and G6 (d) condensates formed in phosphate buffer (pH 7.5) at 20 mM in the presence of 20 mM NaCl or Na2SO4. Boxplots represent 3 independent measurements for G2, G4, and G6 and a single repeat for G3.

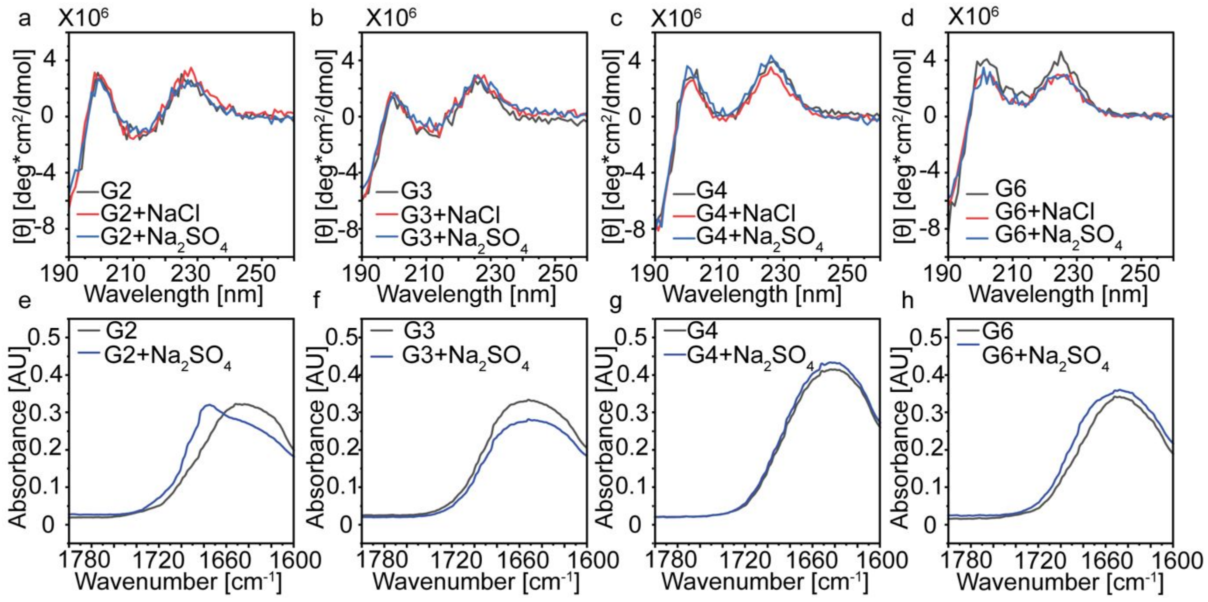

Supplementary Figure 9. CD (**a-d**) and ATR-FTIR (**e-h**) analyses of the peptides G2 (**a, e**), G3 (**b, f**), G4 (**c, g**), and G6 (**d, h**) either in the absence or presence of NaCl or Na_2SO_4. For the CD analysis, samples were prepared at a peptide and salt concentration of 20 mM and then diluted 20-fold to avoid scattering. The ATR-FTIR analysis was performed at peptide and salt concentration of 20 mM, after lyophilization. All samples were prepared in phosphate buffer pH 7.5.

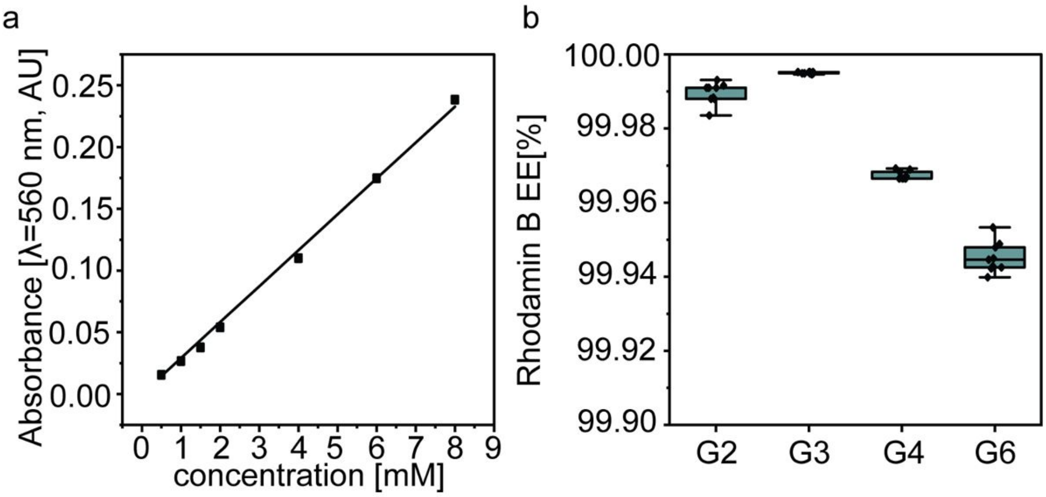

Supplementary Figure 10. a. Absorbance spectroscopy calibration curve of rhodamine b. **b.** Encapsulation efficiency (EE) analysis of rhodamine b (10 μM) in G2, G3, G4, and G6 condensates (20 mM peptide, 20 mM $NaSO_4$) showing the % of dye in the dense phase of each condensate system, based on absorbance spectroscopy λ=560 nm.

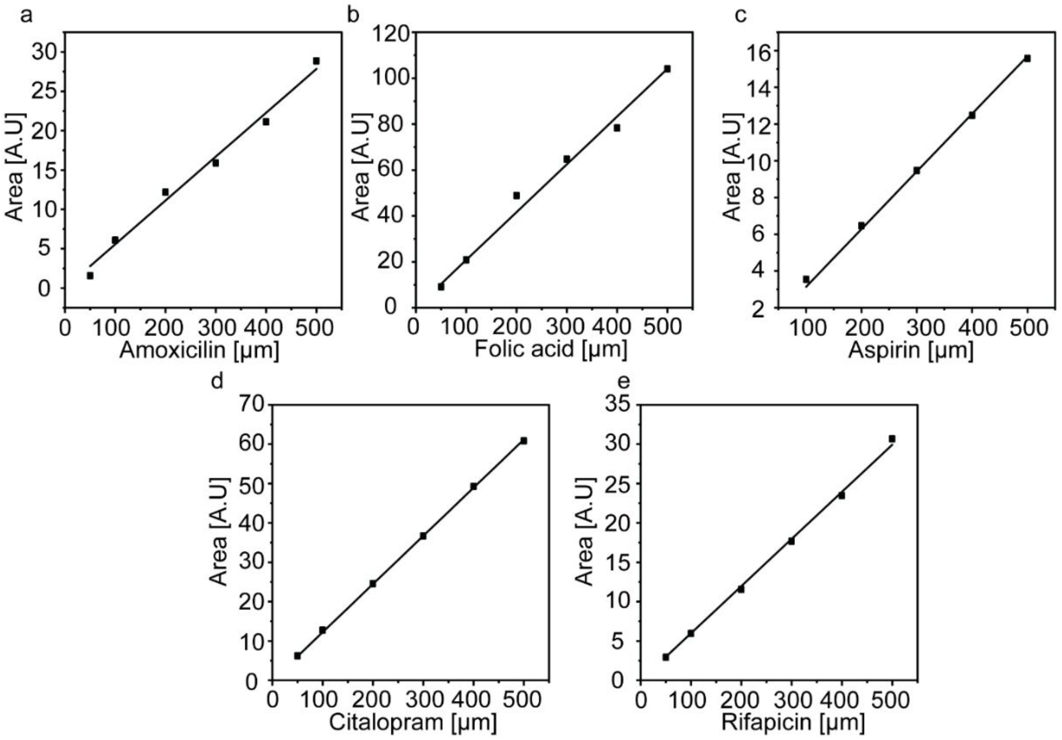

Supplementary Figure 11. HPLC calibration curve of amoxicillin (**a**), folic acid (**b**), aspirin (**c**), citalopram (**d**) and rifampicin (**e**).

**Compounds**	**LogP**	**pKa**	**Charge state at pH=7.5**
Amoxicillin	-0.58	pKa 1= 3.2<br>pKa 2=6.5<br>pKa 3=9.4	~ -1
Folic acid	-0.82	pKa 1=3.5<br>pKa 2=4.1	-2
Aspirin	1.18	pKa=3.3	-1
Citalopram	3.86	pKa=9.9	+1
Rifampicin	3.85	pKa 1=5.3<br>pKa 2=8.3<br>pKa 3=9.3<br>pKa 4=11.9<br>pKa 5=14.1<br>pKa 6=15.9	-1

Supplementary Table 2. Chemical properties of small molecule therapeutic payloads.

	NaCl and Na_2SO_4-free		NaCl		Na_2SO_4	
	EE %	Loading %	EE %	Loading %	EE %	Loading %
Amoxicillin	36.7±6.0	9.2±1.5	44.4±2.7	13.3±0.8	36.2±4.9	1.5±0.2
Folic acid	96.1±1.3	24.0±0.3	95.5±1.4	28.6±0.4	99.7±0.4	4.0±0.0
aspirin	28.3±8.4	7.1±2.1	35.0±4.3	10.5±1.3	36.6±4.8	1.5±0.2
citalopram	24.5±11.6	6.1±3.0	26.6±9.7	8.0±3.0	22.2±8.8	0.9±0.4
Rifampicin	60.1±2.5	15.0±0.6	61.6±2.0	18.5±0.6	78.7±1.2	3.2±0.0

Supplementary Table 3. Encapsulation efficiency [%] of therapeutic compounds in the dense phase of G2 condensates.

	NaCl and Na_2SO_4-free		NaCl		Na_2SO_4	
	EE %	Loading %	EE %	Loading %	EE %	Loading %
Amoxicillin	36.3±5.5	4.2±0.6	33.3±4.0	2.2±0.2	32.4±1.7	1.3±0.1
Folic acid	97.4±0.9	11.3±0.1	96.07±1.0	6.4±0.1	99.7±0.5	3.9±0.0
aspirin	10.1±5.9	1.2±0.7	15.4±6.2	1.0±0.4	14.5±4.2	0.6±0.2
citalopram	26.3±3.3	3.1±0.4	20.6±8.5	1.4±0.6	19.3±9.9	0.8±0.4
Rifampicin	35.8±4.4	4.2±0.5	30.7±3.7	2.0±0.2	64.6±4.4	2.5±0.2

Supplementary Table 4. Encapsulation efficiency [%] of therapeutic compounds in the dense phase of G4 condensates.

	NaCl and Na_2SO_4-free		NaCl		Na_2SO_4	
	**EE %**	**Loading %**	**EE %**	**Loading %**	**EE %**	**Loading %**
Amoxicillin	56.1±2.7	7.0±0.3	52.3±2.5	16.4±0.8	43.6±3.3	2.0±0.2
Folic acid	88.1±1.0	24.0±0.3	86.8±3.1	28.6±0.4	96.3±4.0	4.0±0.0
aspirin	63.1±3.4	7.1±2.1	70.4±1.9	10.5±1.3	59.4±1.3	1.5±0.2
citalopram	9.4±3.7	6.1±3.0	14.2±2.7	8.0±3.0	26.6±14.6	0.9±0.4
Rifampicin	32.1±5.6	4.0±0.7	43.9±10.9	13.7±3.4	65.9±2.9	3.1±0.1

Supplementary Table 5. Encapsulation efficiency [%] of therapeutic compounds in the dense phase of G6 condensates.

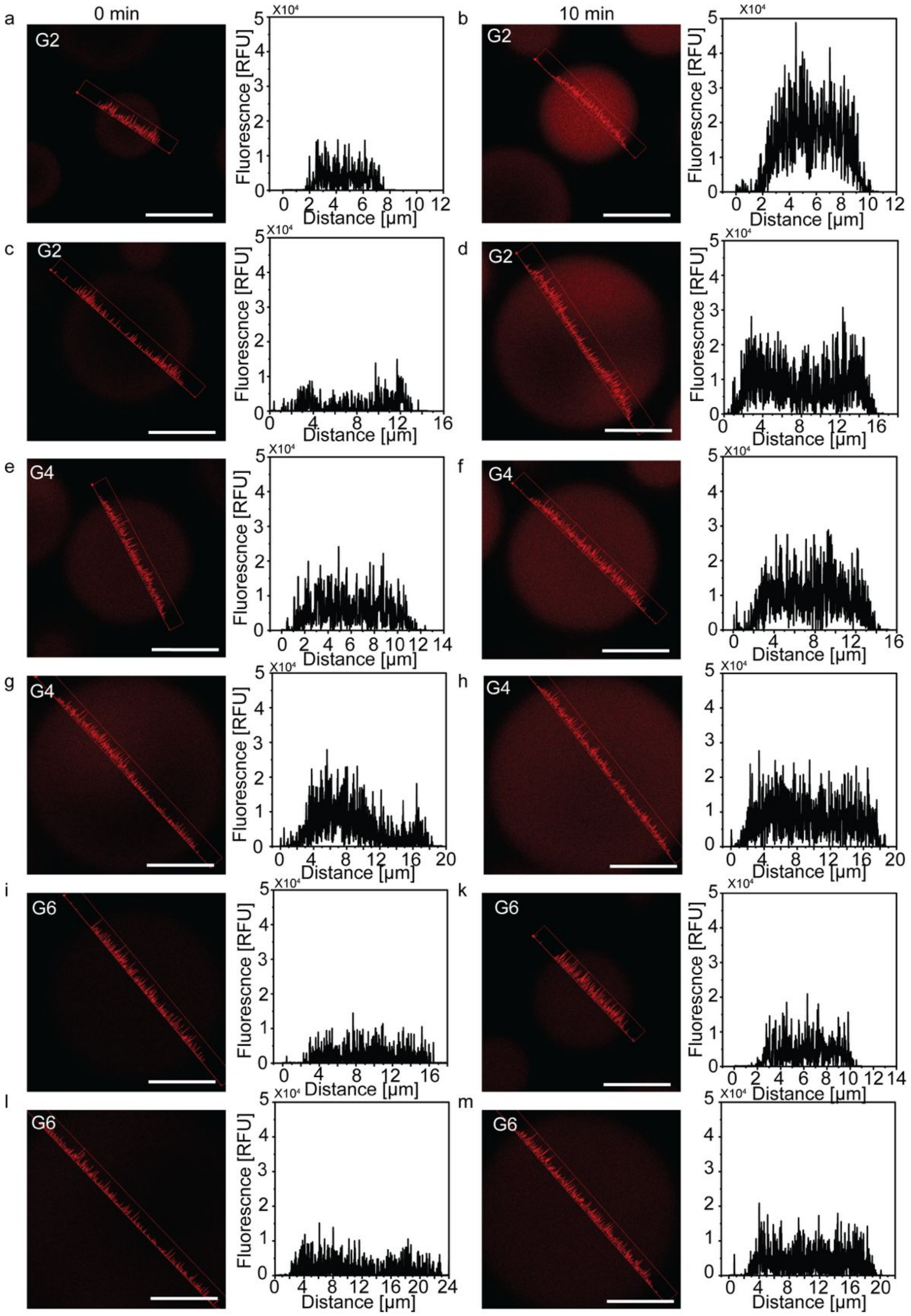

Supplementary Figure 12. CLSM analysis of Atto644-labeled trypsin localization and distribution within peptide condensates. The analysis was performed using excitation laser λex=640 nm and using z-stacking and fluorescence intensity line profiles. For each peptide condensates, images show condensates with small diameter (a-b, e-f, i-k) and large diameter (c-d, g-h, l-m) at t=0 and t=10 min after trypsin addition. Scale bar=5 µm.

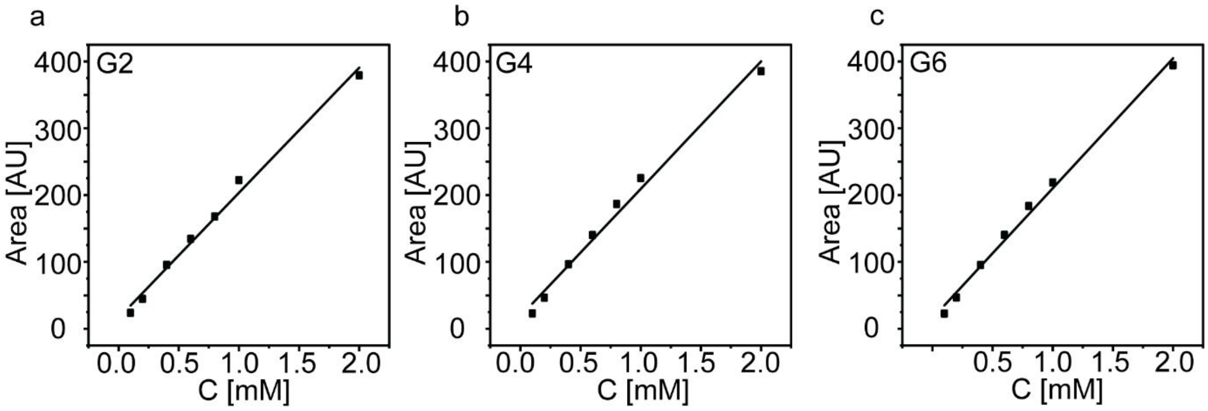

Supplementary Figure 13. HPLC calibration curve of G2 (**a**), G4 (**b**) and G6.

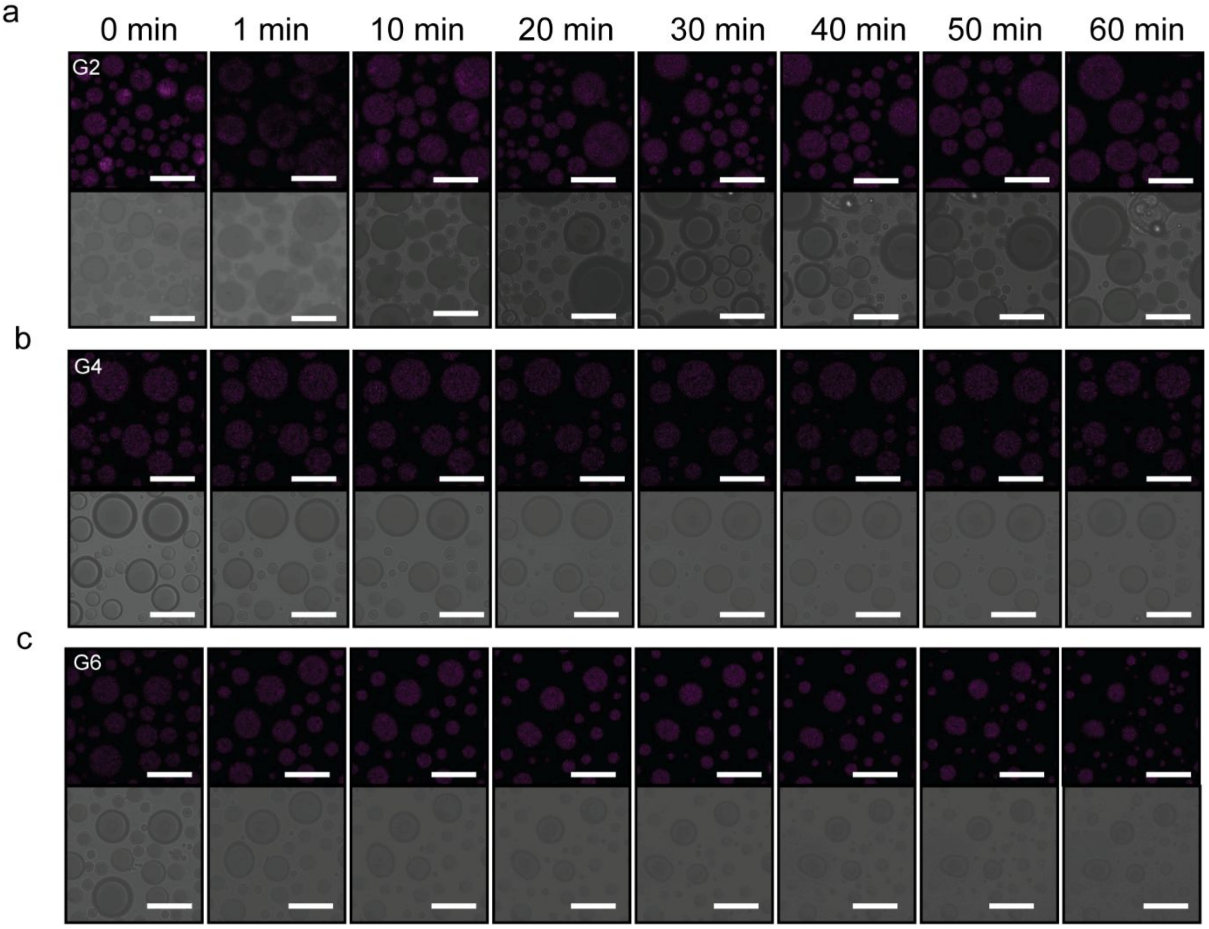

Supplementary Figure 14. CLSM time-lapsed imaging of untreated rifampicin-loaded condensate. Pre-formed condensates loaded with the antimicrobial compound rifampicin (450 μM) were treated with buffer (10% v/v). Condensates formed by 20 mM of G2 (**a**), G4 (**b**), and G6 (**c**), in the presence of 20 mM Na_2SO_4. Scale bar=20 μm.

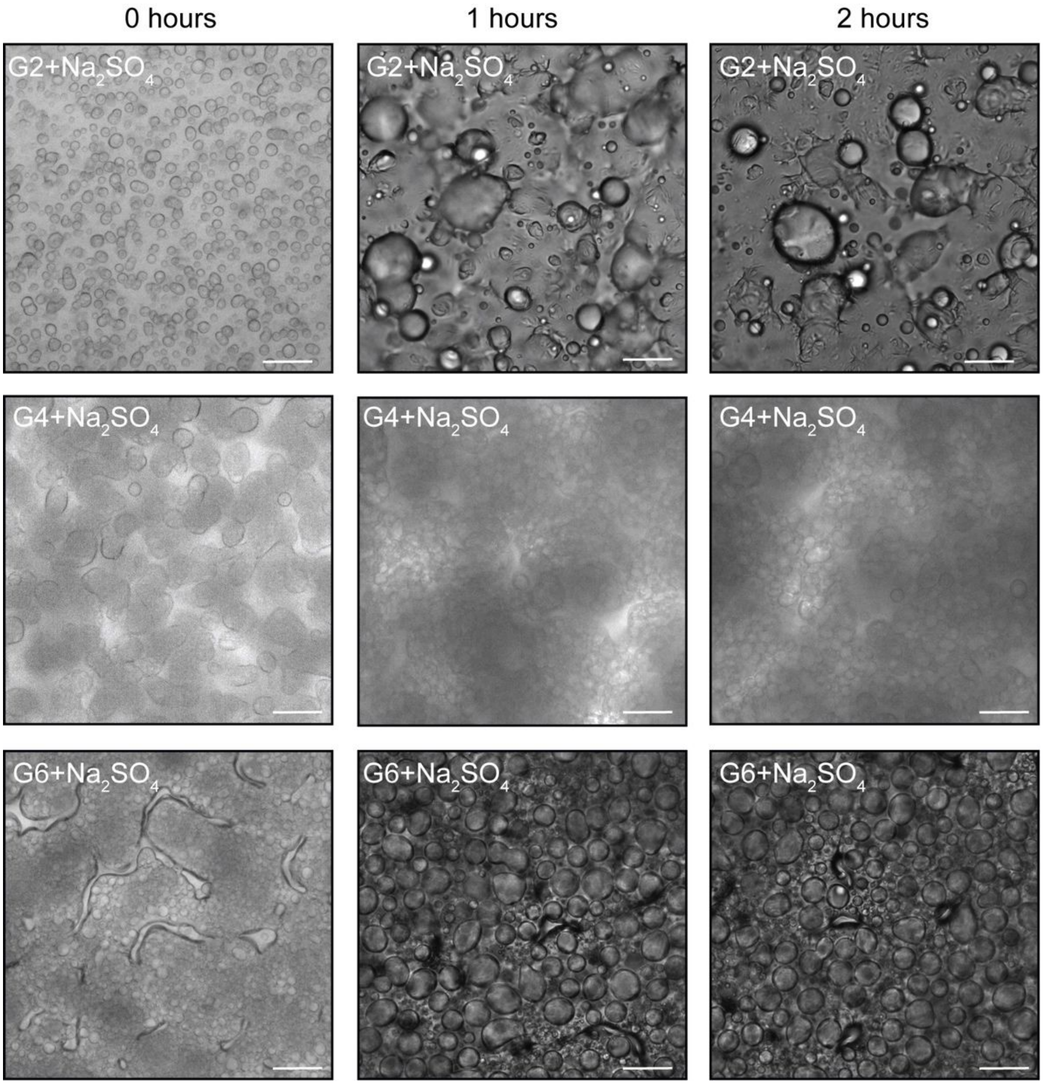

Supplementary Figure 15. Brightfield microscopy analysis of condensates formed by G2, G4, and G6 with Na_2SO_4 in cell culture medium (RPMI) containing 10% FBS serum during the course of 2 h. Scale bars=25 μm.
